# Quantifying generalized trust in individuals and counties using language

**DOI:** 10.3389/frsps.2024.1384262

**Published:** 2024-06-06

**Authors:** Salvatore Giorgi, Jason Jeffrey Jones, Anneke Buffone, Johannes C. Eichstaedt, Patrick Crutchley, David B. Yaden, Jeanette Elstein, Mohammadzaman Zamani, Jennifer Kregor, Laura Smith, Martin E. P. Seligman, Margaret L. Kern, Lyle H. Ungar, H. Andrew Schwartz

**Affiliations:** 1 Department of Computer and Information Science, University of Pennsylvania, Philadelphia, PA, United States; 2 Department of Sociology, Stony Brook University, Stony Brook, NY, United States; 3 Department of Psychology, University of Pennsylvania, Philadelphia, PA, United States; 4 Department of Psychology, Stanford University, Stanford, CA, United States; 5 SonderMind, Denver, CO, United States; 6 Department of Psychiatry and Behavioral Sciences, Johns Hopkins University School of Medicine, Baltimore, MD, United States; 7 Department of Computer Science, Stony Brook University, Stony Brook, NY, United States; 8 Melbourne Graduate School of Education, University of Melbourne, Parkville, VIC, Australia

**Keywords:** generalized trust, social media, social epidemiology, language analysis, data driven approaches

## Abstract

Trust is predictive of civic cooperation and economic growth. Recently, the U.S. public has demonstrated increased partisan division and a surveyed decline in trust in institutions. There is a need to quantify individual and community levels of trust unobtrusively and at scale. Using observations of language across more than 16,000 Facebook users, along with their self-reported generalized trust score, we develop and evaluate a language-based assessment of generalized trust. We then apply the assessment to more than 1.6 billion geotagged tweets collected between 2009 and 2015 and derive estimates of trust across 2,041 U.S. counties. We find generalized trust was associated with more affiliative words (*love*, *we*, and *friends*) and less angry words (*hate* and *stupid*) but only had a weak association with social words primarily driven by strong negative associations with general othering terms (“they” and “people”). At the county level, associations with the Centers for Disease Control and Prevention (CDC) and Gallup surveys suggest that people in high-trust counties were physically healthier and more satisfied with their community and their lives. Our study demonstrates that generalized trust levels can be estimated from language as a low-cost, unobtrusive method to monitor variations in trust in large populations.

## 1 Introduction

In 2017, the Edelman Trust Barometer survey reported an unprecedented international decrease in trust in business, media, government, and non-government organizations (Harrington, 2017). In 2018, the same survey reported another drop in trust in the United States (Friedman, 2018). Imagining low and falling levels of trust leading to negative social, political, and economic outcomes is easy. Therefore, measuring trust consistently, persistently, and at scale is crucial. Here, we take a step in that direction by proposing and applying a machine learning method to transform observable online text data into estimates of generalized trust.

The broadest conceptualization of trust is generalized trust, or one’s expectation of the trustworthiness of others. Generalized trust is responsive to circumstances yet is relatively stable and traitlike (Nannestad, 2008; Dinesen and Bekkers, 2017). Generalized trust influences the wellbeing, prosperity, and health of both individuals and communities (Colquitt and Scott, 2007; Nannestad, 2008; Helliwell and Wang, 2011; Dinesen and Bekkers, 2017). We endeavored to estimate trust with language data at the individual level and use the relationships discovered to infer levels of trust at a population level.

At the individual level, multiple observational and self-report measures of trust exist with very high levels of correlation between one another (Couch and Jones, 1997; Colquitt and Scott, 2007; Glanville and Paxton, 2007; Nannestad, 2008; Delhey et al., 2011; Dinesen and Bekkers, 2017). The most common measure of trust is *the social trust question* (i.e., “Generally speaking, would you say that most people can be trusted or that you can’t be too careful in dealing with people?”; Nannestad, 2008; Eldblom and Jarl, 2012). Generalized trust (hereafter referred to simply as “trust”) is the basic assumption that unknown others are generally good, trustworthy, and more likely to help us than harm us (Yamagishi and Yamagishi, 1994). While other forms of trust concern specific institutions or the government (Dinesen and Bekkers, 2017), generalized trust is about confidence in non-specific others (Rousseau et al., 1998).

At the population level, studies on regional variation in trust have focused almost exclusively on comparisons between countries (Nannestad, 2008; Helliwell and Wang, 2011) and states within the United States (Fairbrother and Martin, 2013). Despite the profound differences in culture between intrastate regions (such as counties) in the United States, little is known about variation in trust at this level. Social scientists have only just begun focusing on counties as a level of analysis, and the only available studies focus on measures of *social capital*—a broader and more contentious category than trust (Rupasingha et al., 2006).

The current research seeks to expand the understanding of trust at individual and community levels through a novel measurement approach. We first develop a language-based assessment of trust using ∼16,000 individuals’ social media language across an average of 19 months as data. This is done by training and validating a machine learning model that predicts trust from language. Next, we apply the trust-based model at the community level (U.S. counties) to explore regional health and behavioral correlates of social trust and distrust. Finally, we explore associations between political partisanship, or fractionalization within communities, and county-level trust.

### 1.1 Prior research on trust

Some scholars view trust as an innate psychological disposition that is rigid (Nannestad, 2008; Van Lange et al., 2014; Dinesen and Bekkers, 2017) or fixed in childhood and not affected by day-to-day experiences later in life (Bowlby, 1969; Couch and Jones, 1997; Uslaner, 1999, 2002). Others believe that trust shifts daily or is impacted by life events and experiences (Rotter, 1971; Hardin, 2002; Glanville and Paxton, 2007; Paxton and Glanville, 2015). Supporting the latter perspective, trust has been found to increase with a breadth of foreign travel experience (Cao et al., 2014) and decrease after job loss (Laurence, 2015) or when frequently moving between neighborhoods within a 5-year period (Helliwell and Wang, 2011). First-generation immigrants moving from a less trusting to a more trusting country display higher levels of trust than do individuals in their countries of origin (Dinesen and Bekkers, 2017). As a whole, it seems likely that generalized trust has both malleable and stable components (Van Lange, 2015; Dinesen and Bekkers, 2017).

Studies across a range of fields indicate numerous individual- and community-level benefits associated with higher levels of trust. At the individual level, trust is positively associated with traits such as social and emotional intelligence, self-esteem, sense of control, attachment security, optimism, tolerance, and acceptance of dissimilar others, as well as lower suspiciousness, jealousy, and shyness (Yamagishi, 2001; Hooghe et al., 2012; Oskarsson et al., 2012; Carl and Billari, 2014; Dinesen and Bekkers, 2017). Trust also predicts a variety of prosocial behaviors, such as civic engagement, volunteering, and integration into one’s neighborhood (Marschall and Stolle, 2004; Helliwell and Wang, 2011; Dinesen and Bekkers, 2017). Personal relationships are positively impacted by higher levels of trust (e.g., Larzelere and Huston, 1980; Simpson, 2007; Murray et al., 2012). In contrast, distrust is associated with poorly differentiated self-concepts, loneliness, social phobia, and Machiavellianism (Couch and Jones, 1997; Simpson, 2007; Rotenberg et al., 2010). Low levels of trust also predict depression, anxiety, and poor physical health (Bienvenu et al., 2004; Helliwell and Wang, 2011; Qualter et al., 2013; Widiger, 2015).

Several studies have considered trust differences across demographic groups. Croson and Buchan (1999) showed that women tend to be less trusting than men, despite women being more trusted (Cappelen et al., 2020). The positive relationship between age and trust has been shown to be robust across several measures of trust and holds across countries (Li and Fung, 2013). It has been suggested that this could be associated with prioritizing social and emotional connectedness later in life or even physical and cognitive decline, as older people tend to rely more on others (Li and Fung, 2013). A computational linguistics framework could reveal these signals through mentions of family, support, events, and even physical/cognitive decline.

At the community level, trust features prominently in several definitions of social capital. Putnam defined social capital as “social networks and the associated norms of reciprocity and trustworthiness” (Putnam, 2007, p. 137; see also Stolle et al., 2008). Trust has been measured as a facet of social capital by the social trust question (Claibourn and Martin, 2000; Grootaert et al., 2004), and there are large differences in social trust across different countries (Halpern, 2005) and states within the United States (Fairbrother and Martin, 2013).

Other work has considered differences in trust across cultural and ethnic groups, comparing individualistic and collectivistic cultures (e.g., Bond and Cheung, 1983; Yuki et al., 2005). At these larger aggregated levels, trust has been named a crucial social attribute in an increasingly globalized world economy, where interactions with unfamiliar others are inevitable (Dinesen and Bekkers, 2017). It is highly predictive of economic growth and more effective governments (Knack and Zak, 2003; Delhey and Newton, 2005), civic engagement (Uslaner and Brown, 2005), and cooperation with strangers (Dinesen and Bekkers, 2017). Trust further correlates with wellbeing and lower rates of suicide (Helliwell and Wang, 2011).

Social, political, ethnic, and economic diversity have been found to decrease generalized trust in some studies—although this effect is moderated by the quantity and quality of social interactions across these intra-community differences (Stolle et al., 2008). One might expect increased immigration into European countries to predict decreases in trust, but a study aiming to measure this relationship found no evidence (Hooghe et al., 2009). Other studies have shown that while immigration can reduce trust, the effect is substantially smaller than other factors, such as economic difficulties and levels of social connectedness (Sturgis et al., 2011). The dramatic shifts in trust in the United States and abroad noted at the beginning of this article may be occurring in response to an increasingly diverse population, or other factors could be at work. Researchers investigating these domestic and international shifts in generalized trust would be greatly aided by the ability to measure trust at larger scales and in shorter time intervals.

The relationship between trust and politics has been shown to be complex and contested. Van Ingen and Bekkers (2015) showed very small or non-significant causal effects between generalized trust and civic engagement despite the two being highly correlated. Although the correlations are robust, work has suggested that trust is not the direct mechanism behind increased engagement, but the increase of both trust and civic engagement seems due to an increase in underlying prosociality (Van Ingen and Bekkers, 2015). Others have found that trust (political and social), at both the individual and community levels, is associated with an increased likelihood of voting and voter turnout (Grönlund and Setälä, 2007; Rahn et al., 2009). It has also been shown that increased welfare programs and social spending are linked to higher levels of trust (Brewer et al., 2014). Other aspects of politics, such as partisanship, also have multifaceted relationships with trust. Hooghe and Oser (2017) showed that partisanship (strength of party affiliation) is positively related to political trust (i.e., trust in political institutions) but negatively linked to generalized trust. This relationship between political trust and partisanship is independent of party affiliation (e.g., Democrats and Republicans) and has remained stable over time. The negative relationship between generalized trust and partisanship shows that partisanship decreases social cohesion, which is in line with literature showing that increased party divisions contribute to polarization. Several of these studies examined trust across larger spatial units, such as countries, and thus, a finer grained county-level analysis may add additional context to this line of research.

Characterizing traits at the regional level is an emerging trend made possible by access to larger data sets and computational techniques. Regional personality traits, measured using a large-scale online survey, were found to have strong associations with social involvement, demographics, crime, religiosity, occupation, and health-promoting behaviors (Rentfrow et al., 2008; Ebert et al., 2022; Giorgi et al., 2022). In the subfield sometimes referred to as “geographical psychology,” regions are thought to differ, in aggregate, across personality and other characteristics based on selective migration patterns. That is, individuals often move to areas in which individuals better match one’s own traits. In some cases, individuals can collectively develop characteristics that are advantageous in a given physical environment (e.g., climate, population density, and the availability of resources; Rentfrow et al., 2008). Social influence—that is, assimilating to the traits that are valued in one’s region—and environmental influence may also lead to “regional traits.”

### 1.2 Measuring trust

Trust has been primarily assessed through experimental observations, self-report scales, and single items in large national surveys (Nannestad, 2008). Lab-based experiments typically involve a task in which individuals can decide whether or not to cooperate (Berg et al., 1995). An illustrative example of experimental research in the field involves the dropped-wallet paradigm, in which a wallet containing personally identifying information and money is dropped in a public place and the rates of wallet return serve as a behavioral measure of trust in communities (Nannestad, 2008; Helliwell and Wang, 2011).

A variety of generalized trust scales exist, such as the Faith in People scale (Rosenberg, 1957) or Yamagishi and Yamagishi’s (1994) Trust Scale. These scales, however, have been criticized for including factors of other constructs (e.g., optimism), combining assessments of one’s own perceived trustworthiness with the trustworthiness of others, and including related but distinct aspects, such as trust in another’s competence (Nannestad, 2008; Dinesen and Bekkers, 2017). Several studies have successfully used the trust items from the Agreeableness factor of Big Five personality questionnaires, as agreeableness reflects an interpersonal approach orientation coupled with a desire to attain closeness with others despite costs to the self, which amounts to a close approximation of trust (Costa and McCrae, 1992; Colquitt and Scott, 2007; Graziano and Tobin, 2017).

In large-scale surveys, self-report measures of trust are also commonly used, ranging from single-item measures that have been included in national (e.g., Gallup, Pew, and the General Social Survey) and international (e.g., European Social Survey, Pew Global, and Our World in Data) surveys for several decades to longer self-report measures for use in smaller studies (e.g., Rotter, 1967; Yamagishi and Yamagishi, 1994; Couch and Jones, 1997). The most widely used assessment of trust is the social trust question, originating from Rosenberg’s (1957) classic Faith in People scale: “Generally speaking, would you say that most people can be trusted or that you can’t be too careful in dealing with people?” To remedy the specificity problem, some researchers have added items asking about the trustworthiness of neighbors, strangers, friends, and other types of individuals directly (Helliwell and Wang, 2011).

In sum, trust has been adequately assessed in behavioral observations, psychometric scales, and single items in large surveys. However, experimental and survey research, when adequately powered, are costly to conduct, in addition to being potentially obtrusive. Recognizing the usefulness of measuring trust but being cognizant of barriers to large-scale measurement, researchers have urged for better assessment methods (Nannestad, 2008; Dinesen and Bekkers, 2017).

### 1.3 The current study

The advent of large online repositories of linguistic data and machine learning methods are now beginning to make measuring trust unobtrusively at scale possible. Automatic language-based assessments using social media data have provided valuable insights into people’s personalities, emotions, and behaviors (Kern et al., 2014; Park et al., 2015; Curtis et al., 2018); identity (Rogers and Jones, 2021); mental health (Preotiuc-Pietro et al., 2015a); wellbeing (Schwartz et al., 2013a, 2016); and physical health (Eichstaedt et al., 2015). On social media, people frequently report their in-the-moment attitudes, feelings, and thoughts about the events that occur in their lives. Once a language model has been trained on scores for a given construct, linguistic patterns can be used to predict scores on that construct over other users using language alone (Schwartz et al., 2013b; Park et al., 2015; Preotiuc-Pietro et al., 2015b). We follow this approach to measure trust based on language shared on Facebook.

The current study examines the possibility of measuring trust at scale by developing and validating a language-based assessment of generalized trust. This is done in five stages. First, we derive a measure of self-reported trust. Second, we explore the language of trust by looking at how language varies across trusting and distrusting individuals, as well as variation across age and gender. Third, we build and validate a language-based machine learning model of trust. Fourth, this model is then applied to ∼6 million geotagged Twitter users, providing a large-scale assessment of trust at the county level (i.e., regions within the United States), which we then validate. Finally, we use the county-level trust measure to gain new insights into community-level health, wellbeing, and political ideology.

## 2 Data

### 2.1 Participants

The analytic sample was drawn from 154,000 users who directly consented to take a personality survey and share their Facebook language data (Kosinski et al., 2015). After consenting, participants completed a Big-Five personality questionnaire based on the International Personality Item Pool proxy for the NEO Personality Inventory (NEO-PI-R; Costa and McCrae, 2008), ranging from 20 to 336 items. Participants could choose to share their own Facebook statuses across the past 4 years for research purposes. Facebook statuses are short autobiographical posts that contain salient personal or emotional information, customarily shared only with individuals designated as “friends.” The use of the de-identified data set, including consenting users only (not their friends), was approved by the University of Pennsylvania’s institutional review board (protocol #813820). The institutional review board deemed this low risk and granted exemption status, including no special requirements for adolescents beyond the same assent that all participants gave. The current study included a subset of users who (a) indicated English as a primary language, (b) completed the 100-item version of the International Personality Item Pool (IPIP) survey (which includes trust-relevant items), (c) indicated their age and gender, and (d) had at least 1,000 words across all of their status updates, as this threshold is common in order to have an adequate sample of each participant’s language (Schwartz et al., 2013b; Kern et al., 2016). The resulting sample included 16,487 users (56.8% female; mean age 23.3 years, *SD* = 9.4 years, range = 13–80) who had shared 3,300,928 Facebook status updates (median status updates = 150) from January 2009 to November 2011.

### 2.2 Individual-level self-report measures

#### 2.2.1 Trust

To measure self-reported trust, we adopted an approach taken by other trust researchers (Colquitt and Scott, 2007; Graziano and Tobin, 2017) by averaging three trust items from the 100-item version of the IPIP personality questionnaire. The trust items are (1) “I believe that others have good intentions,” (2) “I trust what people say,” and (3) “I suspect hidden motives in others” (reverse-coded). These items have strong content validity and have been used successfully in other trust studies (Colquitt and Scott, 2007; Graziano and Tobin, 2017).

We then collected an additional sample of 1,041 participants from Amazon Mechanical Turk to confirm convergent validity with other trust measures. These individuals took four trust questionnaires: (a) the 3 generalized trust items from the 100-item IPIP questionnaire (Cronbach’s alpha in this sample = 0.73), (b) the 10 trust items from the 336-item IPIP measure of the NEO-PI-R (seven additional items beyond 3 trust items; *α* = 0.91), (c) the six items from the Yamagishi generalized trust scale (Yamagishi and Yamagishi, 1994; *α* = 0.87), and (d) the three trust items from the National Opinion Research Center General Social Survey (NORC-GSS; Davis and Smith, 1991; *α* = 0.59).

#### 2.2.2 Other individual-level variables

##### 2.2.2.1 Age

Age was included both as a continuous variable indicating age and, after being separated into terciles, as three binary indicator variables: pre- and early college: 18 years and younger (*n* = 5,472), college-aged: 19–23 (*n* = 5,853), and post-college: 24 and older (*n* = 5,162). The terciles allow for the models to pick up on non-linear relationships.

##### 2.2.2.2 Gender

Gender was available as a self-reported dichotomous variable for male (*N* = 7,120) and female (*N* = 9,367) users.

##### 2.2.2.3 Other demographics not available

Both race and education are suspected to correlate with trust but were only available for a small number of participants within our data. Research has shown that Facebook is widely used by individuals of all races (Pew Research Center, 2015). For a small subsample of the data set, self-reported race was available, indicating a moderate, yet biased, racial diversity (551 White, 131 Asian, 40 Black, 17 other). Education levels are also diverse on Facebook (Pew Research Center, 2015), and we have no reason to believe our sample would differ substantially.

##### 2.2.2.4 IPIP Big 5 Questionnaire

All 16,487 participants completed at least the 100-item version of the IPIP. The 20 items for each domain (extraversion, conscientiousness, neuroticism, openness) were averaged to create composite scores. For agreeableness, the composite score was based on 17 Agreeableness items, excluding the three trust items.

##### 2.2.2.5 Perceived physical symptoms

Three health items indicating physical health (Pennebaker, 1982), physician visits, days sick, and days of inactivity were completed by 846 participants.

##### 2.2.2.6 Orpheus personality questionnaire

The fair-mindedness and self-disclosure subscales of the Orpheus Personality Questionnaire (Rust and Golombok, 2009) were completed by 926 participants. The fair-mindedness subscale assesses impartiality and fairness in decision-making; the self-disclosure subscale assesses self-disclosure and transparency in self-presentation.

##### 2.2.2.7 Satisfaction with Life Scale

The Satisfaction with Life Scale (Diener et al., 1985), a 5-item measure assessing life satisfaction, was completed by 1,229 users.

##### 2.2.2.8 Self-monitoring

The Self-Monitoring Scale (Snyder, 1974), a 25-item scale assessing the degree to which one regulates self-presentation using situational cues, was completed by 1,102 users.

##### 2.2.2.9 Public profile information

In addition to self-reports of personality, we also collected information from consenting users’ public Facebook profiles. Public profile information was available for all 16,487 users. We used this to determine the normalized number of likes from users and the normalized number of tags of others in users’ photos (no other user data was included).

##### 2.2.2.10 Facebook status updates

Facebook status updates were collected from the 16,487 participants, totaling 3,300,928 [more properties of the status updates are discussed in Park et al. (2015)].

### 2.3 County-level data

We gathered publicly accessible data on geographic regions via Twitter and from national surveys (e.g., CDC and Gallup).

#### 2.3.1 Twitter language

We use the County Tweet Lexical Bank (CTLB; Giorgi et al., 2018), which is an open-source data set of county-level language features extracted from a large U.S. county-mapped Twitter corpus. Full details of the data set can be found in Giorgi et al. (2018), but high-level details are described later to aid the reader. A random 10% sample of Twitter language (called the Gardenhose) was collected between July 2009 and April 2014, which was then supplemented with a random 1% sample from May 2014 to February 2015. Individual tweets were geolocated to U.S. counties via self-reported location information in user profiles and latitude/longitude coordinates (Schwartz et al., 2013a). This was done in a way that minimizes the number of false mappings at the expense of the total number of mapped tweets. The total sample contains ∼37.6 billion tweets, of which 1.64 billion English tweets could be geolocated to U.S. counties. Language features (words, phrases, and topics; described later) were first extracted for each user within a county and then averaged (mirroring the process of taking the mean of a survey sample). For this user extraction–county aggregation pipeline, we only consider users with 30 or more posts and counties with 100 or more such users. A total of 2,041 U.S. counties were included.

#### 2.3.2 Gallup

We obtained 2.2 million responses from the Gallup–Sharecare Wellbeing Index between 2009 and 2016. The survey involved a telephone interview using a dual-frame random-digit-dial methodology that included cell phone numbers from all 50 U.S. states. Approximately 1,000 interviews were completed every day from January 2 through December 30, 2012, and 500 were completed every day from January 2, 2013, through December 30, 2016. Questions were chosen such that we predicted a positive association with trust after recoding several variables. Individual responses were averaged to counties. County variables were included in the analysis if a minimum of *N*_*r*_ people responded to a single item. Since all questions in the Gallup-Sharecare Wellbeing index are not available for each year, we adjusted *N*_*r*_ based on the total number of years the question was available (*N*_*r*_ = 200 for <4 years or *N*_*r*_ = 300 for 5-plus years).

#### 2.3.3 Other county-level variables

We drew on information available for U.S. counties to consider demographic and health correlates of trust at the regional level.

##### 2.3.3.1 Demographics

The percentage of females (*N* = 2,041), median age (*N* = 2,041), and log-transformed population density (*N* = 2,041) were taken from the 2010 Census (US Census Bureau, 2010a,b).

##### 2.3.3.2 Socioeconomics

We collected median household income (log-transformed to reduce skewness, *N* = 2,041) from the U.S. Census Bureau’s American Community Survey (US Census Bureau, 2010c). Educational level was based on the percentage of people within the county that completed high school (*N* = 2,041; US Census Bureau, 2010d). The Gini index of income inequality was collected from the 2010–2014 American Community Survey (US Census Bureau, 2010e).

##### 2.3.3.3 Health and wellbeing

Several health and behavioral measures were available (County Health Rankings Roadmaps, 2012): the percentage of people in the county who were obese (*N* = 2,041), excessively drinking (*N* = 1,869), and smokers (*N* = 1,832); the rate of potential life lost (*N* = 2,037); and self-reported health (*N* = 1,924). Life satisfaction (*N* = 1,749) is measured as the average response to the question, “In general, how satisfied are you with your life?” (1 = *very dissatisfied* and 5 = *very satisfied*; estimates are averaged across 2009 and 2010; Lawless and Lucas, 2011).

##### 2.3.3.4 Lifestyle

Based on their current living/marital status, the percentage of people in the county who were married, separated, or same-sex households was indicated (*N* = 2,041; US Census Bureau, 2010f,g).

##### 2.3.3.5 Mental health

Self-reported mentally unhealthy days (out of the last 30 days; *N* = 2,016; County Health Rankings Roadmaps, 2012) were used as a subjective mental health variable.

##### 2.3.3.6 Politics

Presidential election results were gathered for 2000–2016 (Leip, n.d.). In addition to Republican voting percentages, we also look at the difference between Donald Trump’s (2016 election) and Mitt Romney’s (2012 election) vote shares as well as the difference between Trump’s and the average Republican vote share at the previous four presidential elections (2000–2012). Turnout was defined as the total number of votes over the total population (according to the 2010 Census).

##### 2.3.3.7 Donations

Donation data were gathered from the Database on Ideology, Money in Politics, and Elections (Bonica, 2016). Donation partisanship is calculated as the absolute difference in the number of Republican and Democrat donations divided by the sum of donations to both parties. We used donations from 2012 only, as 2016 donation data were not available. Each county needed a minimum of 500 donations to be considered (*N* = 1,655).

##### 2.3.3.8 Disadvantage

The Childhood Opportunity Index (COI) is a composite index, built from 29 U.S. Census variables, designed to identify disadvantaged communities (Acevedo-Garcia et al., 2020). This index is used in real-world settings, such resource allocation and policy decisions (Lou et al., 2023), and is used here to contextualize the effect sizes of the language-based county-level trust measure.

## 3 Methods

We proceed in five stages: (1) develop and validate a measure of self-reported trust, (2) explore linguistic correlates of trusting and distrusting individuals, (3) build and validate a language-based model of trust, (4) estimate and validate county-level trust scores by applying the language model at scale, and (5) explore county-level correlates of trust.

### 3.1 Developing and validating a measure of self-reported trust

The self-reported trust measure is derived from three trust items in the 100-item version of the IPIP personality questionnaire (as described earlier). We assess convergent validity by comparing our measure to other trust measures: the Yamagishi inventory and the NORC-GSS inventory. Next, we correlate the trust measure with external criteria: Big 5 personality, wellbeing and health measures, self-monitoring, and impulsivity. Finally, we explore how trust is related to social media activity, such as likes, friend network size, and tags in photos. All external criteria are described in Section 2.2.2.

### 3.2 Exploring the language of trust

We used both top-down and bottom-up computational linguistic analysis approaches to identify linguistic correlates of trust. A top-down approach uses an established dictionary, or set of words that reflect categories, which are developed *a priori* based on theory. The most commonly used set of dictionaries in psychological science comes from the Linguistic Inquiry Word Count (LIWC) program (Pennebaker et al., 2015). The LIWC is a text analysis application developed to capture multiple psychological dimensions by counting the frequencies of words in a wide variety of categories. These frequencies can then be correlated with other constructs or used as predictors (Pennebaker and King, 1999). We used the LIWC 2015 dictionaries and considered correlations between trust and each category’s frequency.

We also used a bottom-up approach—*differential language analysis* (DLA; Schwartz et al., 2013b)—which identifies language that characterizes generalized trust through semantically similar language clusters. DLA consists of three main steps: linguistic feature extraction, correlational analysis, and visualization, which are described in the following subsections (see Schwartz et al., 2013b; Kern et al., 2016, for further details). Both the top-down and bottom-up approaches use the open-source Differential Language Analysis ToolKit (Schwartz et al., 2017) for linguistic feature extraction, correlation analysis, and visualization.

#### 3.2.1 Linguistic feature extraction

We extract two types of features from each participant’s status updates: (a) *words* and *phrases* and (b) *topics*. Each status update is split into *words*, capturing the oddities of social media language (e.g., misspellings, shortened words, emoticons). Two- and threeword *phrases* (or *n*-grams) are retained if the words within are more likely to appear together than expected by chance (a “collocation”; Kern et al., 2016). To focus on common language and reduce the occurrence of spurious correlations, features are retained only if they are used by at least 10% of participants. The features are encoded as the relative frequency of that word or phrase per user (i.e., the number of times the word or phrase appears out of all word appearances). *Topics* refer to clusters of related words, which have been generated using latent Dirichlet allocation (LDA; Blei et al., 2003), estimated using Gibbs sampling (Gelfand and Smith, 1990) with the MALLET software package (McCallum, 2002) on the complete Facebook data set [the same 2,000 topic set used in Schwartz et al. (2013b) and Kern et al. (2016)].

#### 3.2.2 Correlation analysis

Once extracted, we employ least-squares linear regression to find the correlation between each feature and trust scores, controlling for age, gender, and other non-trust related items of the Agreeableness factor (uncontrolled results can be found in the Supplementary material). This procedure produces tens of thousands of correlations, so we correct for multiple comparisons using a Benjamini–Hochberg false discovery rate (FDR) correction of *p*-values (Benjamini and Hochberg, 1995). We consider coefficients significant if they have a two-tailed *p*-value less than *α* = 0.05 after correction.

#### 3.2.3 Visualization

The final DLA step visualizes the resulting correlations. Words and phrases are combined into a modified word cloud, where the size of the word indicates the strength of the correlation (the coefficient from standardized multiple linear regression) with trust (bigger words have a stronger correlation), and color indicates their frequency of use (gray is low frequency, blue is moderate frequency, dark red is high frequency). For topics, visualizations display the top 15 most prevalent words within a topic, sized according to their posterior likelihood (how often they appear as a representative of the topic).

#### 3.2.4 Linguistic correlations with demographics categories

To understand the differential correlations between language and trust across groups of users, we employed a novel visualization technique. First, we divided the users by (a) gender (male vs. female users), and (b) age (tercile bins). We then repeated the DLA process, controlling for agreeableness and age (for gender) or gender (for age). We separated positive and negative feature correlations and ranked the magnitude of the correlation within each group (i.e., separate correlations of trust for females and males). We next calculated a rank difference score, which indicates how many ranks higher that feature was for female users vs. male users (in the three age bins, each feature’s rank was compared to the average rank in the other two bins). To visualize the results, words with a negative or zero rank difference (i.e., lower or equal rank than the other group or groups) are colored gray and words with a positive rank difference (i.e., higher rank than the other group or groups) are colored green, with darker shades as the rank difference increases. Thus, the colored words are more predictive of trust for the given gender as compared to the other gender (which also means that gender moderates the relationship between the feature and trust).

### 3.3 Establishing a language-based model of trust

To develop a language-based assessment of trust, we used techniques from statistical learning theory based on penalized (ridge) regression (Hastie et al., 2009). Following the well-validated approach of Park et al. (2015), we use three types of information as features (i.e., independent variables) for the statistical model: (1) relative frequencies of the 1- to 3-word phrases, (2) binary-transformed 1- to 3-grams (1 meaning the user used the word/phrase at least once, zero otherwise), and (3) relative frequencies of topics. We extracted these features from the 19,445 users that had three-item trust scores. We note that the features are extracted across the lifetime of posts across each user and, thus, assess trait-level trust for each person.

We used the same feature selection steps as Park et al. (2015), which consist of (1) selecting features with at least a small univariate correlation with the trust score (based only on the training data) and (2) performing a principal components analysis on the remaining features and limiting dimensions to 10% of the number of observations. These steps were performed independently for each of the three types of features (1- to 3-gram frequencies, binary 1- to 3-grams, and topics).

We established model accuracy by evaluating it over a hold-out set of *N* = 438 users that took the longer, 10-item version of trust (a so-called test set; Zamani et al., 2018). The “training set” used to fit the model consisted of the other *N* = 19,445 users. We use the larger three-item trust data for training, as it is often worthwhile to accept some error in the training set (i.e., due to answering fewer trust items) for a larger sample of observations (Schwartz and Ungar, 2015; Kern et al., 2016), while the more reliable less erroneous 10-item trust is used to establish the accuracy of the model. Accuracy is reported as the mean squared error (MSE) and the disattenuated product-moment correlation coefficient between our model’s predictions of trust score and the trust score according to the 10-item questionnaire over the 438 held-out test-set users.

To consider test–retest reliability, we separated time into four periods (time 1: July–December 2009; time 2: January–June 2010; time 3: July–December 2010; time 4: January–June 2011), with an equal number of users (*n* per bin = 2,370).

Finally, we consider convergent and divergent validity by examining the relationship between our trust measure and the facets of agreeableness: trust, morality, altruism, cooperation, modesty, and sympathy. This was done in a sample of 414 individuals who completed the 336-item IPIP.

### 3.4 Applying and validating at scale across U.S. counties

After establishing the validity of the language-based assessment of trust at the individual level, we applied that model to domains and data sets for which collecting generalized trust measures with traditional methods is infeasible, in this case feature sets extracted from location-mapped Twitter data at the U.S. county level (the CTLB, described earlier). This data set contains county-level language features, identical to the features described in Section 3.3: 1- to 3-gram words and phrases, both relative frequencies and binarized, and normalized topic usage (using the same LDA topic model). The language-based trust model described earlier (i.e., a trained regression model) can then be applied to the county-level language features, producing a trust score for each county.

To validate these county trust estimates, we compared our county-level language estimates against the Gallup–Sharecare Wellbeing Index. Items were chosen such that we predicted a positive relationship with trust.

### 3.5 County-level insights

We then correlated the language model-based estimate of community-level generalized trust with other community-level outcomes from large-scale census/survey, demographic, and environmental data. We use the same correlation method as at the individual level: a linear regression between estimated generalized trust as the independent variable and the county-level outcomes as dependent variables. The magnitude of standardized beta represents the effect size of the relationship, and their corresponding *p*-values are corrected for multiple comparisons using the FDR procedure (Benjamini and Hochberg, 1995). As defined previously, these outcomes include sociodemographic information (income, education, etc.), health (percent obese, mortality rates, etc.), and voting trends (percentage voting Republican in the 2016 presidential election as compared to 2012, voter turnout, etc).

## 4 Results

### 4.1 A measure of self-reported trust

The trust measure demonstrated adequate reliability (*α* = 0.73) and was strongly correlated with other trust measures (Table 1), such as the Yamagishi inventory (*r* = 0.77) and the NORC-GSS inventory (*r* = 0.70; correlations with individual items in Supplementary Table S1, including the trust item from Rosenberg’s Faith in People Scale) in a separate sample (*N* = 1,041, mean age = 33.3, 55% female).

For the Facebook sample (*N* = 16,487), Table 2 provides descriptive information for the three items and the other variables available in the data set and correlations between these measures and survey-based, as well as language-based, trust. Here again, we observed strong convergent validity. As expected, trust is positively associated with related constructs such as fair-mindedness and self-disclosure. Among personality traits, the largest association is with overall agreeableness (agreeableness with the trust-facet items removed), although this correlation also demonstrates substantial independence between agreeableness and trust. Emotional stability was also strongly related to trust. Individual-level trust showed expected associations with health (physician visits, days sick) and wellbeing (life satisfaction). Our data also allowed for the exploration of some weak yet interesting associations with social behavior on the platform.

### 4.2 The language of trust

#### 4.2.1 Top-down correlates

Using the top-down computational linguistic analysis approach (LIWC 2015), the categories most associated with trust (see Table 3) were “positive emotions” (most frequent words in corpus: *:), love*, and *good*), followed by the “affiliation” (*love*, *we*, and *friends*) category. Other categories (in descending correlational strength order) were “time” (*now*, *when*, and *back*), “leisure” (*fun*, *Facebook*, and *family*), “work” (*work*, *school*, and *class*), “relative” (*in*, *on*, and *at*), “home” (*home*, *house*, *bed*, *family*, and *room*), “friend” (*friends*, *dear*, *date*, and *contact*), “achieve” (*work*, *first*, and *lost*), and “drives” (*love*, *get*, and *good*).

Distrust (operationalized as lower values on the three-item trust scale) showed overall stronger associations with LIWC categories (i.e., negative correlations between the trust scale and LIWC categories are considered associations between distrust and LIWC categories). It was correlated (in decreasing order) with “anger” (*hate*, *fuck*, and *stupid*), “negative emotions” (*:(*, *hate*, and *bad*), “swear” (*fuck*, *hell*, and *ass*), “sexual” (*fuck*, *gay*, and *sex*), “death” (*die*, *dead*, and *died*), followed by the “body” category (*sleep*, *heart*, *head*, and *face*), negation (*no*, *don’t*, and *can’t*), the “risk” category (*bad*, *wrong*, *stop*, *lose*, and *worse*), “bio” (*life*, *tired*, and *heart*), and personal pronouns (*I*, *you*, *me*, and *your*).

As the LIWC dictionaries are broad categories, this approach can sometimes be opaque due to aggregation. However, a look at the most frequent words within those categories suggests that trusting individuals appear to write more about relationships with others, leisure time activities, and achievement, while those lower in trust express anger and negativity, swear, and discuss bodily matters and problems.

#### 4.2.2 Words and phrases associated with trust

**FIGURE 1:**
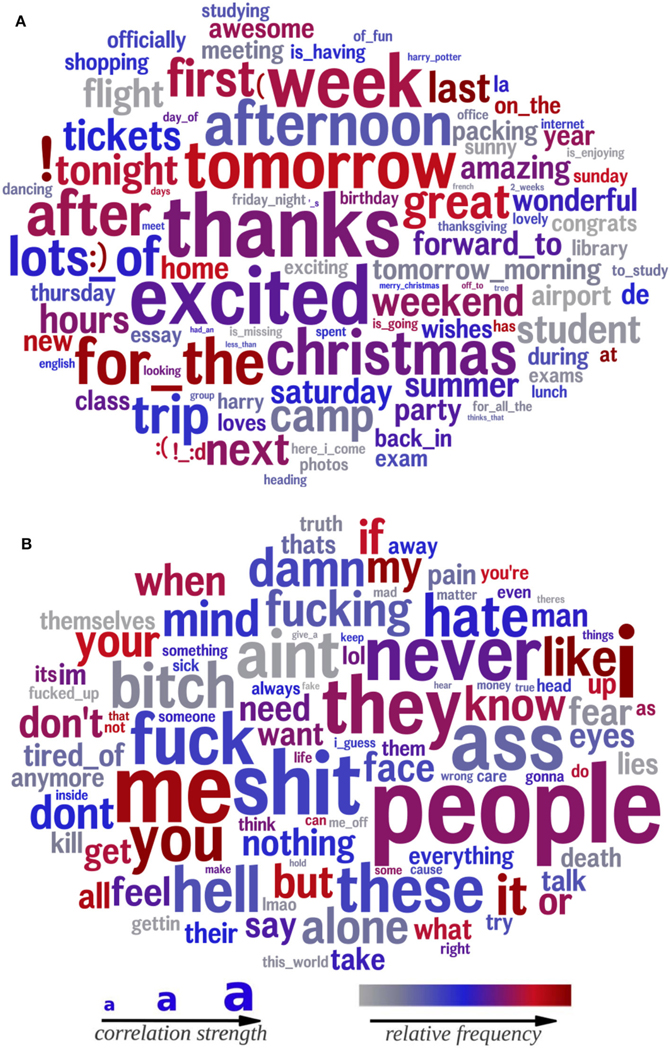
Words and phrases significantly correlated with trust (top; **A**) and distrust (bottom; **B**). Size indicates the relative strength of the correlation (larger = stronger correlation). The color indicates the frequency of use (gray = low frequency, blue = moderate frequency, red = high frequency). Age (terciles), gender, and non-trust agreeableness are controlled for. **(A)** Trust. **(B)** Distrust.

The bottom-up computational linguistic analysis approach yielded similar results. Figure 1 visualizes the words and phrases most strongly related to trust (Figure 1A) and distrust (Figure 1B) based on the DLA. The language of trusting individuals was characterized by positivity (*amazing*, *great*, *awesome*, and *wonderful*), positive anticipation (*next*, *forward to*, and *excited*), and future orientation (*tomorrow*, *afternoon*, *tomorrow morning*, *after*, *tonight*, and *Saturday*). They referenced family and social events (*camp*, *Christmas*, *party*, *tickets*, *meeting*, and *home*), talked about travel (*flight*, *packing*, *camp*, *trip*, and *airport*), and appeared to act prosocially when congratulating others on their accomplishments and giving thanks (*congrats*, *great performance*, *cheers*, and *proud*).

The language of individuals higher in distrust was characterized by greater negativity (*never*, *hell*, *shut*, *fucking*, and *damn*) and aggression (*kill*, *bitch*, and *ass*). The most frequent and highly correlated word of those higher in distrust is “people,” indicating frequent mention of unspecific others and making dehumanized generalizations. Notably, in addition to general negativity, distrusting individuals are negative about people (*hate*, *bitch*, *people*, and *kill*). They appear unhappy, fearful, and lonely (*alone*, *fear*, *pain*, and *tired of* ). They mention trust and honesty directly (*trust*, *truth*, *wrong*, and *lies*) and indirectly (*face* and *eyes*).

**FIGURE 2:**
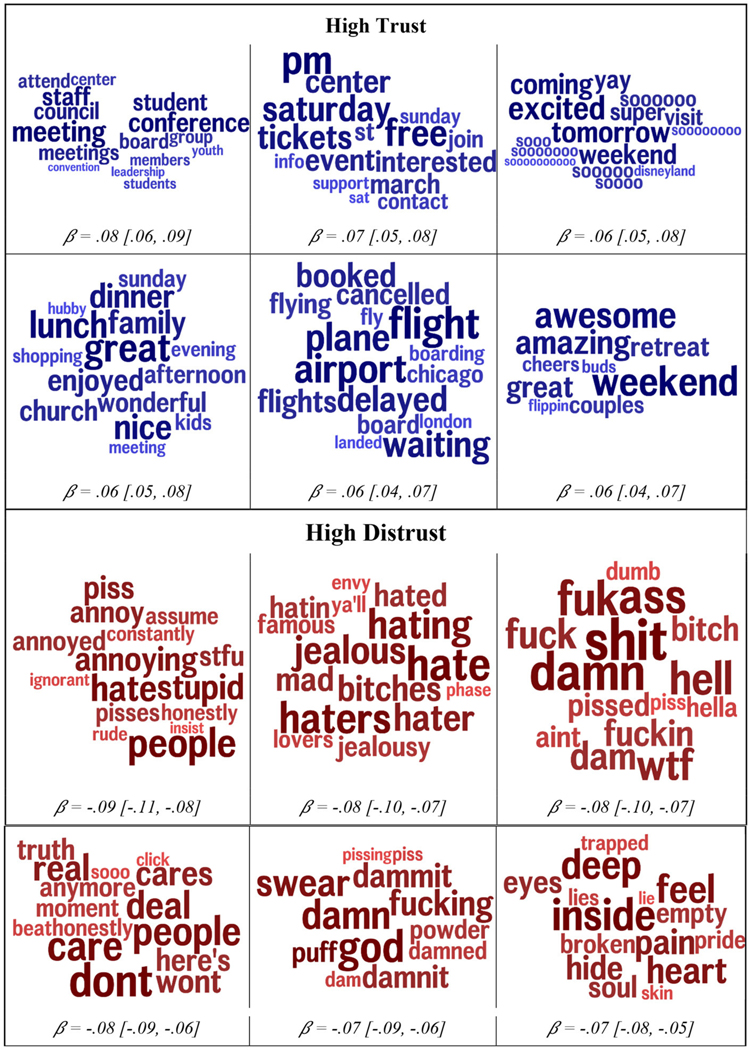
The automatically derived topics that were most strongly correlated with trust **(top)** and distrust **(bottom)**. The size of the word indicates the relative contribution of each word to that topic. The color (darkness) is proportional to size. Age (terciles), gender, and non-trust agreeableness are controlled for. Standardized betas with 95% confidence intervals are reported.

Figure 2 summarizes the topics most strongly correlated with trust and distrust. Supporting the word and phrase patterns, those higher in generalized trust referenced social events (*couples*, *retreat*, and *buds*) and family (*kids*, *dinner*, *family*, and *lunch*), and happy anticipation of future events, many of them social in nature (*Saturday*, *party*, and *weekend*). They referred to not only meals and traveling but also leadership roles (*board*, *council*, and *leadership*).

Topics for distrust again reflected the single-word correlations. Those higher in distrust swore more, especially at or about others. The topics are generally negative in nature and express views of others as stupid, annoying, rude, ignorant, and uncaring (*don’t care*). The word *people* is strongly correlated with low trust—it may be the case that referring to people en masse often reflects negative attitudes (e.g., “people are so rude”), whereas positive attitudes are more localized to persons or smaller communities.

Importantly, these words and phrases are used primarily to build language models for the purpose of prediction, yet the content of these correlates provides a rich source for hypothesis generation. We conjecture that the reference to planning, tickets, marches, and events may indicate that those higher in trust function as the planners and social engineers of their groups, taking a leadership role in their social worlds.

##### 4.2.2.1 Investigating the relationship between trust and social language

**FIGURE 3:**
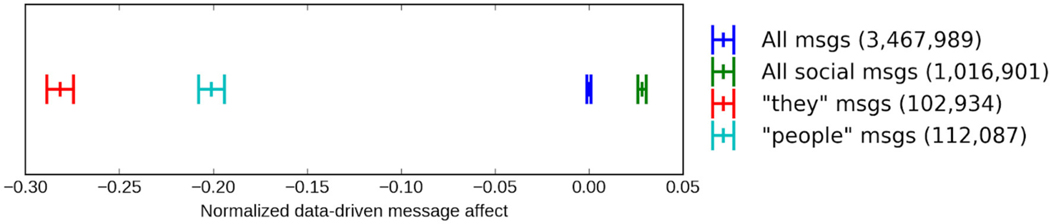
Mean affect score of messages containing, from left to right, *they* (red), *people* (light blue), all messages (blue; the baseline), and any of the 12 Linguistic Inquiry Word Count social words including *people* and *they* (green). The larger bars to the left and right of each indicate a 95% confidence interval.

Given that trust is characterized by more social behavior, we would expect the LIWC “social” category to be strongly associated with trust, which it is not (see Table 3). Therefore, we investigated the presence of the social words *people* and *they* as a marker of distrust, suspecting their use is more common in a negative context. Might those low in trust use *people* as a way to express their misanthropy, like in “*those* people”? We automatically computed an affective score for each message (Preotiuc-Pietro et al., 2016), normalized across the full corpus, and then selected a subset of the LIWC “social” category. If a message contained one of these words, it was tagged as a “social” message, while those containing the word *people* were tagged as “people” messages, and those containing the word *they* are tagged as “they” messages (note that “people” and “they” messages are subsets of all social messages). We calculated the mean affect of all social messages, as well as the subset of social messages containing *people* and *they*. As illustrated in Figure 3, social messages tended to be slightly more positive on average. However, the subset of those social messages that included *people* or *they* were significantly more negative in affect, demonstrating that the words *people* and *they* are more commonly used in negative social messages.

We then create two versions of the LIWC “social” category: (1) one with *people* and *they* words alone and (2) one with *people* and *they* words removed. These two categories are then correlated with self-reported trust in an analysis identical to that in Section 4.2.1. LIWC “social” with *people* and *they* words alone correlated with trust at *β* =−0.10 (−0.11, −0.08), *p* < 0.001, while “social” with *people* and *they* words removed correlated with trust at *β* =−0.01 (−0.02, 0.01), *p* = 0.34. Thus, people who use *people* and *they* less scored higher in generalized trust. Social words other than *people* and *they* show no relationship with trust.

#### 4.2.3 Language of trust across gender and age

**FIGURE 4:**
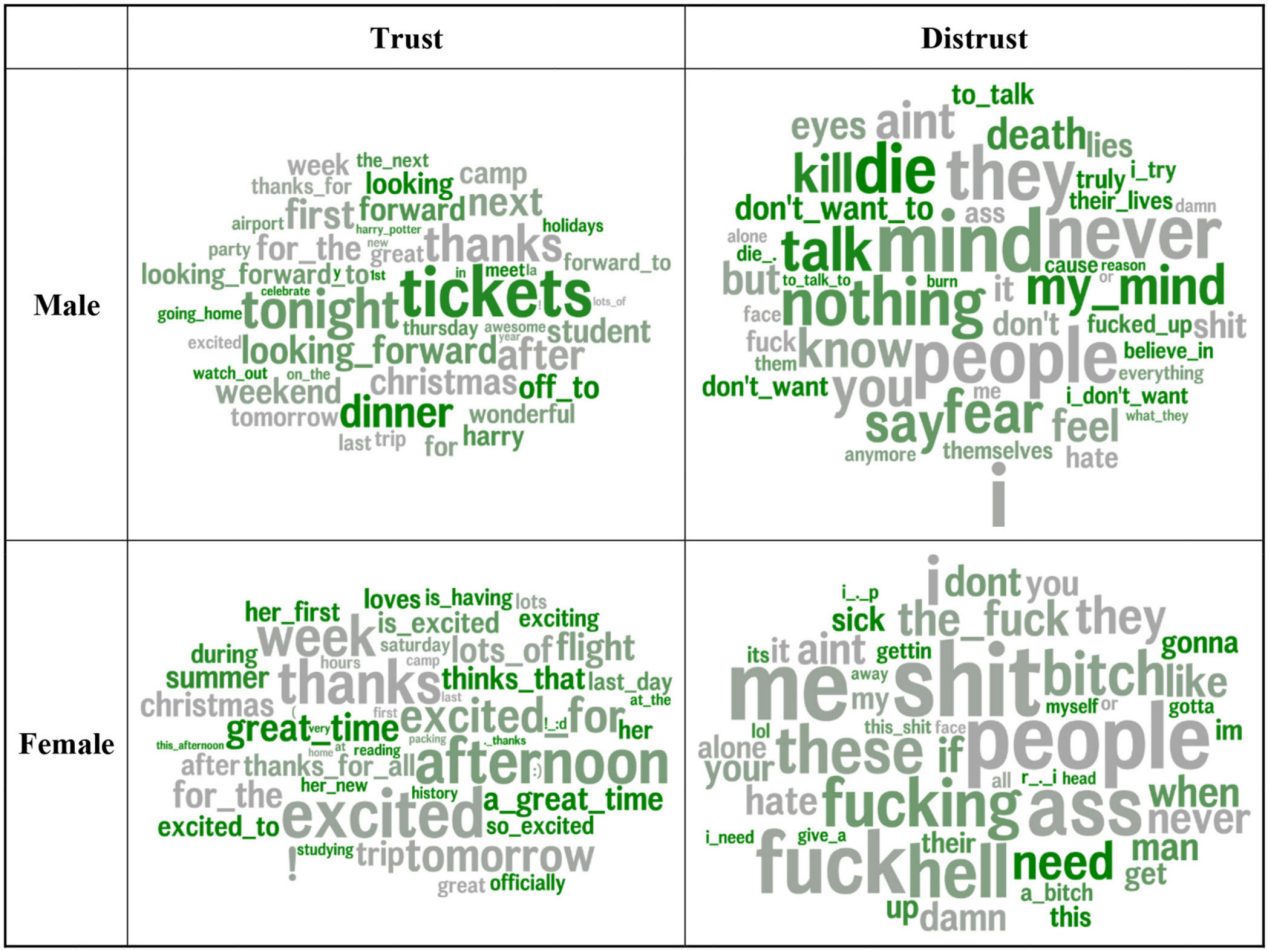
Word and multi-word phrases that were correlated most strongly with trust by gender. Word size corresponds to correlation magnitude. Features are colored by difference in effect size rank vs. other gender. Greener words (e.g., *tickets* for high-trust male users or *need* for low-trust female users) have relatively stronger correlations with trust compared to the correlation strength in the other gender. Gray words (e.g., *people* in low-trust and *thanks* in high-trust for both females and males), have similar correlation strengths across genders. Age and non-trust agreeableness are controlled for.

Next, we examined the language of trust across gender and age groups. Figure 4 illustrates the words and phrases used more by trusting and distrusting males and females. The gray words indicate the similarities across gender in the language that distinguishes trust and distrust. The green words indicate a greater gender difference. Trusting males spoke of social plans and events (*tickets*, *meet*, *dinner*, and *off to*) and positive anticipation of future events (*tonight* and *looking forward*). Trusting females reflect positive sentiment (*great time* and *excited*) in anticipation of (*excited to*), during (*is having*), and as a result of events (*great time* and *a great time*) and speak of learning (*studying*).

Distrusting males are characterized by aggression (*kill*, *die*, and *death*) and apathy (*don’t want to* and *nothing*). Distrusting females express need (*need*, *when*, and *I need*). Notably, distrusting females tend to appear more masculine in their language, as swearing on social media tends to be indicative of masculinity (Schwartz et al., 2013b; Park et al., 2016).

**FIGURE 5:**
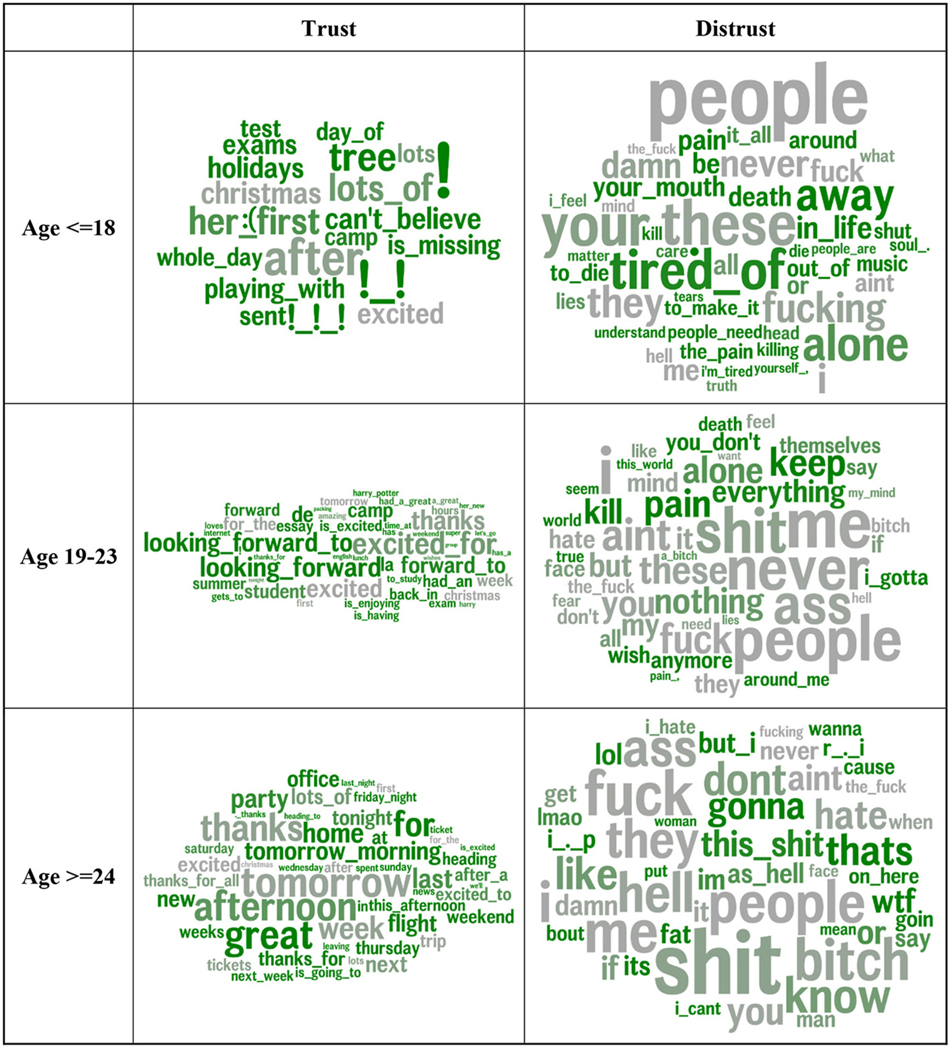
Word and multi-word phrases most strongly correlated with trust by age tertile. Word size corresponds to correlation magnitude. Features are colored by difference in effect size rank vs. other age bins. Green indicates relatively higher effect size rank in that age compared to the average of the other two bins. Gray words have similar correlation strengths across age groups. Gender and non-trust agreeableness are controlled for.

Figure 5 distinguishes trusting and distrusting language across age groups. Words and phrases in gray are similarly associated with trust in all age groups, while those in green indicate a higher effect size rank in that age group compared to the average of the other two age groups. Expressions of gratitude (*thanks*), excitement, and general future orientation (*tomorrow*) were associated with trust in all age groups. *People* consistently correlated with distrust, with a similar rank across the age categories. Younger trusting users expressed wonder (*can’t believe*), emotions (*emojis*), and arousal (*!* and *!!!*), holidays (*tree* and *holidays*), and school (*test*, *exams*, *English*, and *science*). Trusting individuals aged 19–23 were distinguished by positive anticipation (*looking forward* and *looking forward to*). Trusting individuals older than 23 mentioned places (*home* and *office*), travel (*flight*, *ticket*, and *heading*), and social events (*party*).

Younger distrusting individuals noted loneliness (*alone*), confusion (*don’t understand*), and feeling bad (*pain*, *worst*, and *i’m tired*, *to die*). Distrusting individuals aged 19–23 spoke of aggression (*kill*) and emptiness (*pain* and *nothing*). The 24-andolder age group expressed profound negativity [*this shit*, *wtf* (“*what the fuck*”), and *the hell*].

The word size corresponds to correlation magnitude. Features are colored by the difference in effect size rank vs. the other gender. Greener words (e.g., *tickets* for high-trust male users or *need* for low-trust female users) have relatively stronger correlations with trust compared to the correlation strength in the other gender. Gray words (e.g., *people* in low-trust and *thanks* in high-trust females and males) have similar correlation strengths across genders. Age and non-trust agreeableness are controlled for.

### 4.3 A language-based model of trust

To assess the model accuracy, we compare the language-based trust estimates to self-reported trust in the test set. Results show that the model is accurate at MSE = 0.66 and product-moment correlation =0.49 (0.42, 0.56). These results are in line with accuracies predicting psychological constructs from social media text. Park et al. (2015) showed correlations between 0.35 and 0.43 between language-based personality estimates and self-reported personality.

For test–retest reliability, results are shown in Table 4 and were comparable to longitudinal correlations for other personality characteristics (Park et al., 2015). Scores were strongly correlated across the time points (range = 0.57–0.74), with decreasing strength over time.

In terms of convergent and divergent validity (i.e., comparing our trust measures to facets of agreeableness), the language-based measure was very strongly correlated with trust (*β*_survey_ = 0.77) and moderately correlated with overall Agreeableness (*β*_survey_ = 0.53), with the strongest correlation with the trust facet, and the weakest correlation with modesty (*β*_survey_ = 0.12) and mortality (*β*_survey_ = 0.12; see Table 5).

### 4.4 Validating a county-level trust assessment

**FIGURE 6:**
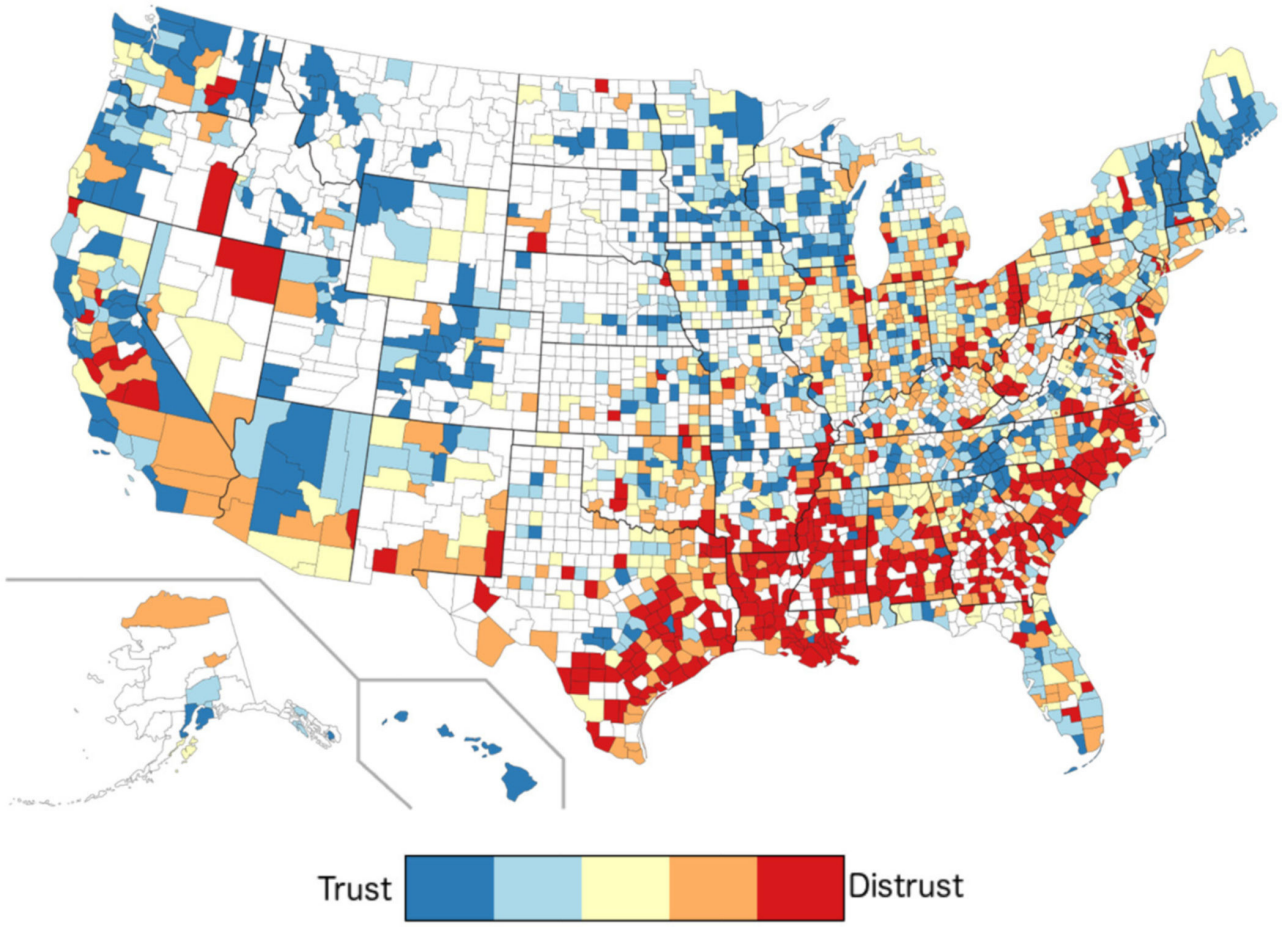
Levels of trust by U.S. counties. Red indicates higher levels of distrust; blue indicates higher levels of trust. White counties did not have sufficient Twitter language information available.

We applied the Facebook-derived model to geotagged Tweets and mapped them to U.S. counties (see Figure 6), with higher trust in blue and lower trust in red. Strong regional variations emerge (see Supplementary Figure S5 for a map in which sex, age, race, population, log density, and log income are controlled for).

We compared our county-level language estimates against the Gallup–Sharecare Wellbeing Index. Items were chosen such that we predicted a positive relationship with trust. Table 6 shows the items with their predicted and actual relationships with trust. All but two items (*Someone in your life encourages you to be healthy* and *Hours spent socially*) matched our predictions.

### 4.5 County-level trust correlates

Further county-level analyses (see Table 7) revealed that trust was associated with more education (greater rate of high school graduation), higher income, and greater population density. More trusting counties had a greater percentage of individuals living in committed, stable relationships, as indexed by higher marriage and same-sex household rates, while less trusting counties had a greater percentage of separated individuals. This mirrors findings at the individual level (Helliwell and Wang, 2011). Areas with more Evangelical Protestants were lower in trust, while areas with more mainline Protestants and Catholics were more trusting, again confirming previous findings at the individual level (see Supplementary Table S3; Welch et al., 2004). Counties with more income inequality showed lower trust, in agreement with previous findings (Kawachi et al., 1997). These results suggest that trusting communities are generally more affluent, educated, and religious. Importantly, trust is low in counties with ethnic, economic, and political diversity.

Strong links to health and wellbeing were evident as well. Trusting counties had better self-rated mental and physical health, confirming what has previously been observed at the state level (Abbott and Freeth, 2008). Trust was also associated with healthier lifestyles (including obesity and percentage of smokers) and greater longevity. Unlike individual-level research that found social capital to be protective against excessive drinking (Takakura, 2011), counties with greater rates of excessive drinking tend to be higher in trust. Counties with greater trust also enjoy greater life satisfaction (congruent with findings at the individual level; Helliwell and Wang, 2011).

Finally, we examined county trust in relation to county political factors (see Table 8). Trusting counties had higher voter turnout in the 2012 and 2016 presidential elections. Distrusting counties had a higher Trump vote *gain* vs. both the Romney vote (2012) and the average Republican vote in the previous four elections (2000, 2004, 2008, and 2012). Donation partisanship (more donations associated with a single party) was associated with more trust.

## 5 Discussion

Trust is a central topic in contemporary discourse on a number of social, political, and economic issues. To better understand and unobtrusively measure trust, we combined self-report questionnaires and linguistic data from Facebook to build a language-based assessment to measure trust at scale. We provided insights into the psychological characteristics of trusting and distrusting individuals and observed the correlational profile of trusting and distrusting communities. We demonstrated that social media language can be used to understand and measure trust and that the measurement can be scaled to estimate the trust of large populations.

Methods for estimating regional traits can be derived from combining text and psychometric data at an individual level, as demonstrated here. Spontaneous, in-the-moment reports of a person’s thoughts and behaviors (e.g., social media posts) are a rich source of data. Such assessment provides emotional, cognitive, behavioral, and personality correlates of a construct quickly and inexpensively by revealing the words, phrases, and topics associated with one’s score on a given construct. Here, we gained a broader, more accurate understanding of trust as a trait, as well as generated hypotheses regarding what may cause shifts in generalized trust. Our method can be used to complement traditional methods by providing researchers with a tool to measure trust at scale.

### 5.1 Individual-level correlates of trust

Consistent with prior research, age and gender correlations with trust were relatively weak (Feingold, 1994; Helliwell and Wang, 2011). The positive association found with fair-mindedness is congruent with the previously suggested association between trust in others and one’s own trustworthiness (Ben-Ner and Putterman, 2001; Hardin, 2004). Consistent with prior research, those higher in trust were higher in subjective wellbeing (Helliwell and Wang, 2011) and reported fewer sick days and physician visits, suggesting that more trusting individuals also may be healthier.

We also explored the association of trust with Facebook behaviors. Users may “tag” other users in posts being shared (including images and posts referencing social events). We found that more trusting individuals “tagged” more people and had more “friends,” perhaps suggesting that trusting users are comfortable sharing personal content with more people.

### 5.2 The language of trust

Associations with LIWC 2015 categories provide a parsimonious overview of the language of trusting individuals, suggesting that those higher in trust express more positive emotions and reference more interpersonal affiliations. They seem to be both engaged in their social and professional lives, referencing not only work life as well as achievement but also relaxation, home, and family time. LIWC results are in line with trusting individuals leading relatively balanced lives. Those lower in trust express anger and negativity, swear, and discuss risk. Of particular note, greater generalized trust was not associated strongly with greater use of social words, in contrast with previous work suggesting trusting individuals are more social (Hardin, 2002). However, upon closer inspection, it turned out that the lack of association with social terms was driven by the negative effect of othering terminology (*they* and *people* were associated with distrust) that countered positive associations with other social words (e.g., *we*). This motivates the idea of observing open-vocabulary analyses that do not make *a priori* assumptions about words and group membership (Schwartz et al., 2013b).

Open-vocabulary approaches yielded more specific language associations. We found that trusting individuals mentioned travel (*flight*, *packing*, and *airport*), supporting recent evidence that a breadth of travel (number of times traveled) is associated with generalized trust (Cao et al., 2014). Trusting individuals appeared integrated into cohesive social networks due to their more frequent mention of them (family, kids, and couples), which seemed to involve initiating or participating in social outings (*free*, *pm*, *interested*, and *march*) and taking an optimistic, happy, and future-oriented outlook on life (*excited* and *tomorrow*) when approaching others. Trusting individuals appeared to be more socially engaged (*meeting*, *event*, *board*, and *council*) and prosocial (*amazing*, *great*, and *cheers*), perhaps taking an active role in making a positive difference for their social networks, communities, or causes they care about, for example, being a council member and attending meetings. This suggests the benefits of trust not just for the individual but also for employers and the greater community through positive effects on health, wellbeing, and overall flourishing.

The language of distrusting individuals was different, marked by misanthropy (*stupid*, *annoyed*, *people*, and *bitches*), hatred (*hate*, *hating*, and *hatin*), and swearing (*fuck*, *damn*, and *ass*) about or at others, but it was also indicative of social isolation, emotional pain, and sadness (*pain*, *broken*, *inside*, and *empty*), suggesting that these negative views of others are deeply upsetting and alienating. It is possible that those who experience rejection or alienation from others (e.g., bullying, discrimination, and rejection) develop lower levels of generalized trust. As to be expected, individuals low in trust also appeared to be preoccupied with others’ flaws, such as lying and selfishness (*eyes*, *truth*, and *lies*), and to frequently experience conflict and discord in their relationships with others (*people*, *don’t care*, and *rude*). A focus on the self is also characteristic of those low in trust (*I* and *I’m*).

Distrusting adolescents mentioned loneliness, disappointments, and feeling misunderstood, as well as thoughts about pain and death. During early adulthood, for those low in trust, pain topics still prevailed but were joined by expressions of not caring (*anymore*) and some aggression, while post-college aggressive sentiment dominates along with swearing. From adolescence into young adulthood, distrust appeared to shift from being more self-focused to being more other-focused, suggesting that individuals low in trust might perceive themselves as the cause for their discord with others and then blame others and become disillusioned later in life. These patterns align with theory and research suggesting that the younger years are more important for the formation of trust (Erikson, 1963; Inglehart, 1997; Putnam, 2000) before pain and disappointment morph into disengagement and anger. Those lower in trust also mentioned the words *people* and *they* more, which tends to be mentioned more in negative contexts. This holds for distrusting males and females (Figure 4) and all age groups (Figure 5). Following the construal level theory of psychological distance (Trope and Liberman, 2010), it is possible that those lower in trust think more often about people as abstract and larger collectives, while those higher in trust are more prone to focusing on individuals.

Future research could directly explore the malleable aspects of generalized trust (Glanville and Paxton, 2007). It would be interesting to examine changes in the level of trust within individuals over time, both as a function of key life events and as impacted by changes in the individual’s social network. Whereas such an analysis would be almost impossible with self-reported information, requiring an intensive longitudinal design, the temporal and network-based structure of social media can allow such a dynamic analysis. Using multilevel modeling approaches, it would be possible to identify the factors that lead to the rise or demise of the social trust of individuals and communities, individual–community fit based on trust, and the success of interventions aimed at increasing social trust. A deeper understanding of how social trust is negotiated, maintained, reduced, and increased as a function of time will be crucial for determining how social trust can be improved both for individuals and for communities.

### 5.3 Correlates of county-level trust

County-level correlations (see Table 7) showed that more affluent areas and those with greater income equality are higher in trust, consistent with prior research (Nannestad, 2008). Trusting communities were also generally healthier and happier than distrusting communities, suggesting the importance of social cohesion and cooperation on regional health. A culture of mutual trust and respect may affect the health of its residents in a myriad of ways, from relying on health care and increasing one’s family income to visiting doctors and following doctor’s orders to taking advantage of public parks and other spaces to socialize and exercise. It has been suggested that building communities that are safe, walkable, and encourage positive social interactions may benefit both the sense of trust and the economic, physical, and mental wellbeing of the community (Jackson, 2003; Mason, 2010).

A notable exception of these positive effects of generalized trust at the community level is seen in a positive association with excessive drinking, a generally socially negative health behavior (Kuntsche et al., 2017). While it is possible that some third variable explains this relationship [although we controlled for socioeconomic status (SES) and region], it is plausible that trust may make people more comfortable to drink, as they are less concerned about interpersonal risks or that trusting communities celebrate together more. The role of alcohol in forming social bonds and trust is worth further study.

Otherwise, areas with greater distrust were those at greater risk of being unhealthy and premature death. It might be that these communities are marked by infrastructural deficits that do not allow for easy access to social interaction (e.g., long distances to recreational centers for social activity and exercise coupled with poor walkability and access to public transportation, a lack of social events, and a lack of safe, public places for gathering; Cohen and Inagami, 2005; Diez Roux and Mair, 2010) or that a culture of negativity makes it unattractive to spend time being active and interacting with others (Eichstaedt et al., 2015). This would be in line with suggestions that designing public spaces to make walking and socializing with others attractive will encourage embeddedness in social groups and promote health and longevity (Buettner, 2008).

Finally, Table 7 shows the relationship between the COI and the county-level outcomes. This index (and similar disadvantage indices) is currently used to allocate resources and guide policy decisions (Lou et al., 2023). The effect sizes for the COI are slightly larger, which is to be expected because this is a composite index built from 29 U.S. Census variables, as opposed to a language-based psychological construct. Nevertheless, the magnitude and direction effect sizes for the language-based trust measure and the COI are comparable, showing that our trust measure could be used in similar real-world settings to help develop effective interventions or even guide policy decisions. For example, the language of highly trusting individuals contains references to traveling, community events, and time with friends and family. This may suggest that for trust to be built, individuals may need to connect both with their own community more as well as travel outside of it, although more research is needed to draw firm conclusions on this point. Furthermore, continuous monitoring through language assessment could be deployed in aid to those who wish to reverse the general decrease in trust in the United States. Best practices for fostering trust across social, economic, and political divisions need to be established, shared, and tested. While causality has not been addressed here, the correlations suggest that fostering trust may well be important for improving the health and prosperity of communities. This may be particularly the case in highly fractionalized communities.

### 5.4 Politics and trust

In the United States, confidence in institutions has been on the decline over the past several decades (Gallup, 2022). One way to examine the implications of this trend is to consider the relationship between trust and voter preference. We found the relationship between trust and party preference shifted between the 2012 and the 2016 presidential elections. The correlation between trust and Republican vote share moved away from the positive toward the negative. More aligned were the county trust levels and the votes *gained* by the 2016 Republican candidate (Donald Trump) over previous Republican candidates. Trump was supported more in counties that used language indicative of high distrust.

Donald Trump’s election win was surprising to many (Flegenheimer and Barbaro, 2016). Early work to explain the result employed postelection surveys and focused on demographic segments (especially the “white working class”), ascribing motives based on personal identity (Morgan and Lee, 2018; Schaffner et al., 2018). The prospective data-driven approach employed here, however, enabled a window into trust based on verbal behavior during the campaigns. Together, the open-vocabulary method and large social media language data set covered orders of magnitude more people and more open-ended themes for hypothesis testing. Importantly, the data are longitudinal, providing data before, during, and after the election.

Two possible hypotheses follow from the pattern of results. First, communities lower in generalized trust prefer populist candidates (those that attempt to appeal directly to voters and circumvent parties, the media, and other institutions) to establishment candidates. Second, decreasing levels of generalized trust lead to lower vote shares for incumbent and incumbent-party candidates. These predictions are speculative—based on one surprising election result, but the real-time and local nature of the social media data could allow for future evaluations to be tested in a principled way—by predicting which type of candidate will be unexpectedly popular both locally and nationally.

### 5.5 Limitations

Several limitations of this study should be acknowledged. First, at the time of data collection, social media users tended to be younger than the general population (Duggan and Smith, 2013), and, accordingly, most participants in our sample were young adults (median age = 20, range = 13–80). Still, we are able to control for age in our analyses, and due to the overall size of our data, we still have more participants 30 and older (*N* = 3,354) than most samples in trust research, which primarily occurs with university students.

Second, the current article is based on a survey-based measure of trust that utilizes trust items taken from the NEO-PI-R (Costa and McCrae, 1992). This scale is one of the most established measures of trust and has been used in previous research (Colquitt and Scott, 2007; Graziano and Tobin, 2017) and was highly correlated with other established trust scales. However, it is possible that if we fit our language-based model to a different scale, then it would have slightly different properties. Trust research has been criticized in general for its flawed assessment (e.g., Glaeser et al., 2000; Evans and Revelle, 2008; Nannestad, 2008), and a social media approach provides a valid alternative for future research that reduces problems with traditional self-report survey measures, including recency effects, recall bias, difficulty accessing diverse populations, and cost (Park et al., 2015; Vazire, 2015).

Third, a key question and potential limitation for all online behavior-based research is whether it reflects their offline personality and whether behavior online is consistent with offline behavior. Research establishing the validity of social media methods has demonstrated that there is a good indication that users tend to present themselves accurately to their social network (Kosinski et al., 2013).

Fourth, we identified a variety of correlates at the individual and community levels. While the multi-test corrections to significance give confidence to the reliability of these results, they do not suggest causal relationships. Other studies, using a variety of methodologies and samples, would replicate and refine the pattern of results revealed through our analyses using more targeted hypotheses.

## 6 Conclusion

Trust is a topic at the center of contemporary social, economic, and political discourse. In this study, we systematically identified reliable correlations between individuals’ language and their scores on traditional generalized trust scales. Additionally, we introduced a social media language-based assessment of trust that can be used to estimate the level of trust or distrust within communities. This measure demonstrated meaningful regional variation across U.S. counties. This work provides insights into how individuals higher vs. lower in generalized trust differ, suggests a method to consistently and persistently monitor trust in large populations, and identifies key correlates of trust at the community level.

## Supplementary Material

Giorgi S, Jones JJ, Buffone A, Eichstaedt JC, Crutchley P, Yaden DB, Elstein J, Zamani M, Kregor J, Smith L, Seligman MEP, Kern ML, Ungar LH and Schwartz HA (2024) Quantifying generalized trust in individuals and counties using language. Front. Soc. Psychol. 2:1384262. doi: 10.3389/frsps.2024.1384262_SupplementaryMaterials

Supplementary material

The Supplementary Material for this article can be found online at: https://www.frontiersin.org/articles/10.3389/frsps.2024.1384262/full#supplementary-material

## Figures and Tables

**TABLE 1:** Convergent validity: correlations with the three-item trust measure.

	Statistics						*r*
	*N*	Mean	SD	Min	Max	Skew	
Yamagishi inventory	1,041	3.18	0.82	1	5	−0.26	0.77 [0.74, 0.79]
NORC-GSS inventory	1,041	6.05	1.55	1	11	−0.03	0.70 [0.67, 0.72]

Separate Amazon Mechanical Turk survey sample (N = 1,041; mean age=33.3, 55% female). Product-moment correlation is reported with 95% confidence intervals in brackets. All results are significant at p < 0.001 after adjusting for multiple comparisons. NORC-GSS, National Opinion Research Center General Social Survey.

**TABLE 2:** Descriptives and correlations with the three-item trust measure in the Facebook sample.

		Statistics						*β*
		*N*	Mean	SD	Min	Max	Skew	
Facebook usage	Number of likes from user	16,487	232.31	406.77	0	4,597	4.68	−0.04 [−0.05, −0.02]
	Number of tags in users’ photos	16,487	114.13	187.79	0	3,081	3.41	0.08 [0.06, 0.09]
	Number of status updates	16,487	211.55	180.17	9	1,936	2.70	−0.03 [−0.04, −0.01]^▲^
	User word total	16,487	4,248.02	3,998.28	1,000	58,979	3.36	−0.02 [−0.03, −0.00]^▲^
	Demographic network size	16,487	226.18	238.60	0	4,329	4.51	0.08 [0.06, 0.09]
Demographics	Age	16,487	23.35	9.44	13	80	2.49	0.06 [0.04, 0.07]
	Gender (0 = male, 1 = female)	16,487	0.57	0.50	0	1	−0.28	0.08 [0.06, 0.09]^†^
Personality	Extraversion	16,487	3.40	0.78	1.00	5.00	−0.30	0.26 [0.24, 0.27]
	Agreeableness*	16,487	3.65	0.60	1.06	5.00	−0.53	0.53 [0.52, 0.54]
	Conscientiousness	16,487	3.42	0.68	1.10	5.00	−0.20	0.13 [0.12, 0.15]
	Neuroticism	16,487	2.74	0.79	1.00	5.00	0.21	−0.37 [−0.38, −0.35]
	Openness	16,487	4.00	0.51	1.15	5.00	−0.57	0.03 [0.01, 0.04]^▲^
Orpheus Personality	Fair-mindedness	926	1.73	6.13	−16.50	17.50	−0.11	0.23 [0.16, 0.29]
	Self-disclosure	926	0.16	6.73	−16.00	17.50	−0.01	0.23 [0.17, 0.29]
Wellbeing/health	Self-reported days sick	846	4.09	9.18	0	99	5.54	−0.10 [−0.17, −0.03]^▲^
	Self-reported number of visits to physician	846	0.70	2.38	0	45	11.10	−0.10 [−0.16, −0.03]^▲^
	Self-reported number of days of restricted activity due to illness	846	3.18	10.58	0	99	6.43	−0.06 [−0.12, −0.01]^†^
	Satisfaction With Life Scale	1,229	4.23	1.43	1.20	6.80	−0.18	0.32 [0.27, 0.37]
Other scales	Snyder’s Self-Monitoring	1,102	8.39	3.50	0.00	21.00	0.39	−0.07 [−0.12, −0.01]^†^
	Barratt Impulsivity Scale Score	771	1.42	1.07	0.00	3.17	−0.45	0.01 [−0.06, 0.08]^†^

All variables other than age and gender are controlled for age (terciles) and gender. Standardized betas are reported with 95% confidence intervals in brackets. All results are significant at p < 0.001 after adjusting for multiple comparisons, except those marked with

▲ where p < 0.05, and

† which are not significant.

* Based on the 17 Agreeableness items, excluding the three trust items.

**TABLE 3:** Significant correlates of trust and distrust with Linguistic Inquiry and Word Count categories (*p* < 0.001, adjusted for multiple comparisons).

Trust			Distrust		
Category	Words	*β*	Category	Words	*β*
Posemo	:), love, good, happy, lol	0.11 [0.10, 0.13]	Anger	Hate, fuck, stupid, hell, sucks	−0.22 [−0.23, −0.20]
Affiliation	Love, we, friends, our, facebook	0.08 [0.07, 0.10]	Negemo	:[, hate, bad, miss, sick	−0.19 [−0.21, −0.18]
Time	Now, when, back, new, then	0.08 [0.06, 0.09]	Swear	Fuck, hell, ass, sucks, crap	−0.18 [−0.20, −0.17]
Leisure	Fun, facebook, family, play, playing	0.07 [0.06, 0.09]	Sexual	Fuck, gay, sex, sexy, dick	−0.14 [−0.16, −0.13]
Work	Work, school, class, working, read	0.06 [0.05, 0.09]	Death	Die, dead, died, alive, war	−0.13 [−0.15, −0.12]
Relative	In, on, at, up, out	0.06 [0.04, 0.07]	Body	Sleep, heart, head, face, ass	−0.11 [−0.13, −0.10]
Home	Home, house, bed, family, room	0.06 [0.04, 0.07]	Negate	Not, no, don’t, can’t, never	−0.10 [−0.12, −0.09]
Friend	Friends, friend, dear, date, contact	0.05 [0.04, 0.07]	Risk	Bad, stop, wrong, worse, lose	−0.09 [−0.11, −0.08]
Achieve	Work, best, first, better, lost	0.05 [0.03, 0.06]	Bio	Love, life, sleep, tired, heart	−0.08 [−0.09, −0.06]
Drives	Up, love, get, we, good	0.05 [0.03, 0.06]	Ppron	I, you, my, me, your	−0.08 [−0.09, −0.06]

Words ordered by descending frequency within the Facebook corpus. All variables are controlled for age (terciles) and gender. Reported standardized betas with 95% confidence intervals in square brackets. All results are significant at p < 0.001 after adjusting for multiple comparisons. Posemo, positive emotion; Negemo, negative emotion; Ppron, personal pronoun.

**TABLE 4:** Cross-time correlations of individual-level language-based assessments of trust.

	*N*	Time 2	Time 3	Time 4
Time 1	2,370	0.68 [0.66, 0.70]	0.64 [0.62, 0.66]	0.57 [0.54, 0.60]
Time 2	2,370		0.74 [0.72, 0.76]	0.64 [0.62, 0.66]
Time 3	2,370			0.68 [0.66, 0.70]

Time 1: July–December 2009; Time 2: January–June 2010; Time 3: July–December 2010; Time 4: January–June 2011. Product-moment correlations are reported with 95% confidence intervals in brackets.

**TABLE 5:** Product-moment correlation with facets of agreeableness.

	Statistics						Trust	
							Survey	Language
	*N*	Mean	SD	Min	Max	Skew	*β*	*β*
Trust	414	3.27	0.87	1.00	5.00	−0.36	0.77 [0.73, 0.81]	0.35 [0.26, 0.43]
Morality	414	3.69	0.71	1.20	5.00	−0.66	0.36 [0.27, 0.44]	0.12 [0.02, 0.21]^▲^
Altruism	414	3.94	0.70	1.20	5.00	−0.78	0.44 [0.35, 0.51]	0.18 [0.08, 0.27]
Cooperation	414	3.40	0.74	1.50	5.00	−0.29	0.51 [0.44, 0.58]	0.28 [0.19, 0.36]
Modesty	414	3.17	0.76	1.20	4.80	−0.29	0.12 [0.02, 0.22]^▲^	−0.02 [−0.12, 0.07]^†^
Sympathy	414	3.56	0.72	1.20	5.00	−0.57	0.41 [0.32, 0.48]	0.06 [−0.04, 0.15]^†^

Controlled for age (terciles) and gender. Reported standardized beta with 95% confidence intervals in square brackets. All results are significant at p < 0.001 after adjusting for multiple comparisons except for those marked with

▲ where p < 0.05, and

† which are not significant.

**TABLE 6:** Gallup–Sharecare Wellbeing Index and county-level language-based trust.

	Gallup		Trust		Assessment	
	Years	*N*	*r*	*N*	Predicted	Actual
Someone in your life encourages you to be healthy	2013–2016	200	−0.14 [−0.21, −0.06]	620	+	–
The city or area where I live is a perfect place for me	2013–2016	200	0.59 [0.53, 0.64]	620	+	+
In the last 12 months, you have received recognition for helping to improve the city or area where you live	2013–2016	200	0.25 [0.18, 0.33]	620	+	+
I can’t imagine living in a better community than the one I live in today	2014–2016	200	0.55 [0.49, 0.61]	476	+	+
I am proud of my community or the area where I live	2014–2016	200	0.59 [0.53, 0.65]	476	+	+
I always feel safe and secure	2014–2016	200	0.51 [0.44, 0.57]	476	+	+
Hours spent socially	2009–2016	300	−0.14 [−0.19, −0.08]	1,225	+	–
Are you satisfied or dissatisfied with the city or area where you live?	2009–2016	300	0.58 [0.54, 0.62]	1,225	+	+
Safe walking alone	2009–2013	200	0.57 [0.52, 0.67]	1,351	+	+
Safe place to exercise	2009–2013	200	0.44 [0.39, 0.48]	1,351	+	+

Reported product-moment correlation with 95% confidence intervals in square brackets. All results are significant at p < 0.001 after adjusting for multiple comparisons. Gallup N is the minimum number of individual responses needed per county; Trust N is the number of counties meeting our minimum language and Gallup responses threshold.

**TABLE 7:** County-level correlations between language-based trust and sociodemographics.

	Year	*N*	No adjustments	Adjusted for region	COI
			*r*	*β*	*β*
**Demographics**					
Percent female	2010	2,041	−0.07 [−0.12, −0.03]	0.01 [−0.04, 0.05]^†^	0.02 [−0.02, 0.06]^†^
Median age	2010	2,041	0.06 [0.01, 0.10]^▲^	0.06 [0.02, 0.10]^▲^	0.03 [−0.01, 0.07]^†^
Log population density	2010	2,041	0.08 [0.04, 0.13]	0.15 [0.11, 0.19]	0.26 [0.22, 0.30]
**Socioeconomics**					
Log income	2010	2,041	0.41 [0.37, 0.45]	0.33 [0.29, 0.37]	0.77 [0.75, 0.78]
Percentage of high school graduates	2005–2009	2,041	0.54 [0.51, 0.57]	0.42 [0.39, 0.46]	0.63 [0.69, 0.65]
Gini	2010–2014	2,036	−0.14 [−0.18, −0.10]	−0.05 [−0.09, −0.00]^†^	−0.33 [−0.37, −0.29]
**Health and wellbeing**					
Year potential life lost rate	2012	2,037	−0.56 [−0.59, −0.53]	−0.43 [−0.47, −0.40]	−0.71 [−0.73, −0.69]
Self-rated health, percent fair/poor	2012	1,924	−0.50 [−0.54, −0.47]	−0.41 [−0.44, −0.37]	−0.65 [−0.67, −0.62]
Percentage obese	2012	2,041	−0.57 [−0.59, −0.53]	−0.46 [−0.49, −0.42]	−0.57 [−0.60, −0.54]
Percentage smokers	2012	1,832	−0.31 [−0.34, −0.26]	−0.22 [−0.26, −0.18]	−0.48 [−0.51, −0.44]
Percentage of excessive drinking	2012	1,869	0.31 [0.27, 0.35]	0.15 [0.10, 0.19]	0.29 [0.25, 0.33]
Life satisfaction	2009–2010	1,749	0.30 [0.25, 0.34]	0.33 [0.29, 0.37]	0.53 [0.50, 0.57]
**Lifestyle**					
Percentage married	2005–2009	2,041	0.24 [0.20, 0.28]	0.23 [0.19, 0.27]	0.49 [0.45, 0.52]
Percentage separated	2005–2009	2,041	−0.52 [−0.55, −0.49]	−0.40 [−0.44, −0.37]	−0.52 [−0.54, −0.48]
Percentage of same-sex households	2005–2009	2,041	0.25 [0.21, 0.29]	0.22 [0.18, 0.26]	0.20 [0.16, 0.24]
**Mental health**					
Mentally unhealthy days	2012	2,016	−0.23 [−0.27, −0.19]	−0.17 [−0.21, −0.13]	−0.41 [−0.45, −0.38]

Product-moment correlations and standardized betas are reported with 95% confidence intervals in brackets. All results are significant at p < 0.001 after adjusting for multiple comparisons except for those marked with, Controls: region [binary indicator for four Census regions: Northeast, South, West, and Midwest]; the Childhood Opportunity Index (COI) column also adjusted for region.

▲ where p < 0.01, and

† which are not significant.

**TABLE 8:** County-level correlations between language-based trust and politics.

	Years	*N*	No Adjustments	Adjusted for region
			*r*	*β*
Turnout	2012	2,032	0.34 [0.30, 0.38]	0.32 [0.28, 0.36]
	2016	2,031	0.46 [0.43, 0.49]	0.45 [0.42, 0.49]
% Republican votes in presidential election	2012	2,032	0.02 [−0.03, 0.06]^†^	0.15 [0.10, 0.19]
	2016	2,031	−0.08 [−0.12, −0.03]	0.03 [−0.01, 0.08]^^†^^
Trump vote gain vs. Romney vote	2012–2016	2,031	−0.27 [−0.31, −0.23]	−0.30 [−0.34, −0.26]
Trump vote gain vs. past 4 Republicans	2000–2016	2,028	−0.15 [−0.19, −0.10]	−0.14 [−0.18, −0.10]
Donation partisanship	2012	1,665	0.19 [0.15, 0.23]	0.12 [0.06, 0.15]

Product-moment correlations and standardized betas are reported with 95% confidence intervals in brackets. All results are significant at p < 0.001 after adjusting for multiple comparisons except for those marked with, Control: region (binary indicator for four Census regions: Northeast, South, West and Midwest).

† which are not significant.

## Data Availability

The data analyzed in this study is subject to the following licenses/restrictions: due to the sensitive and potentially identifying nature of Facebook language data, we are unable to share the individual-level data. The county-level trust estimates are available at: https://osf.io/ap8rx/. Requests to access these datasets should be directed at: SG, sal.giorgi@gmail.com.
