# Supplementary material for: Quantifying generalized trust in individuals and counties using language
Source: Front Soc Psychol. Author manuscript; Available in PMC 2025 Nov 6. (PMC12588161; doi:10.3389/frsps.2024.1384262)
Supplement: Giorgi S, Jones JJ, Buffone A, Eichstaedt JC, Crutchley P, Yaden DB, Elstein J, Zamani M, Kregor J, Smith L, Seligman MEP, Kern ML, Ungar LH and Schwartz HA (2024) Quantifying generalized trust in individuals and counties using language. Front. Soc. Psychol. 2:1384262. doi: 10.3389/frsps.2024.138426 [file NIHMS2112064-supplement-Giorgi_S__Jones_JJ__Buffone_A__Eichstaedt_JC__Crutchley_P__Yaden_DB__Elstein_J__Zamani_M__Kregor_J__Smith_L__Seligman_MEP__Kern_ML__Ungar_LH_and_Schwartz_HA__2024__Quantifying_generalized_trust_in_individuals_and_count.docx]

**Supplemental Material for “Quantifying Generalized Trust in Individuals and Counties Using Language”**

Salvatore Giorgi ^1^, Jason Jeffrey Jones ^2^, Anneke Buffone ^1^, Johannes C. Eichstaedt ^3^, Patrick Crutchley ^4^, David B. Yaden ^5^, Jeanette Elstein ^1^, Mohammadzaman Zamani ^2^, Jennifer Kregor ^1^, Laura Smith ^1^, Martin E. P. Seligman ^1^, Margaret L. Kern ^6^, Lyle H. Ungar ^1^, H. Andrew Schwartz ^2^

^1^University of Pennsylvania, Philadelphia, Pennsylvania, USA

^2^Stony Brook University, Stony Brook, New York, USA

^3^Stanford University, Stanford, California, USA

^4^SonderMind, Denver, Colorado, USA

^5^Johns Hopkins University School of Medicine, Baltimore, MD, USA

^6^University of Melbourne, Parkville, Victoria, Australia

**Table S1**

*Individual NORC items with questionnaire-based trust*

|  | Statistics | | | | | |  |
| --- | --- | --- | --- | --- | --- | --- | --- |
|  | *N* | Mean | Std Dev | Min | Max | Skew | *r* |
| Generally speaking, would you say that most people can be trusted or that you can't be too careful in dealing with people? | 1041 | 5.90 | 2.57 | 1 | 11 | -0.16 | .71 [.68, .74] |
| Would you say that most of the time people try to be helpful, or that they are mostly just looking out for themselves? | 1041 | 6.01 | 2.31 | 1 | 11 | -0.20 | .62 [.58, .66] |
| Do you think most people would try to take advantage of you if they got a chance, or would they try to be fair? | 1041 | 5.76 | 2.14 | 1 | 11 | 0.09 | .18 [.12, .24] |

*Note*: Separate Mturk survey sample (N=1,041; mean age=33.3, 55% female). Reported Pearson *r* with 95% confidence intervals in square brackets. All results significant at p < .001 after adjusting for multiple comparisons.


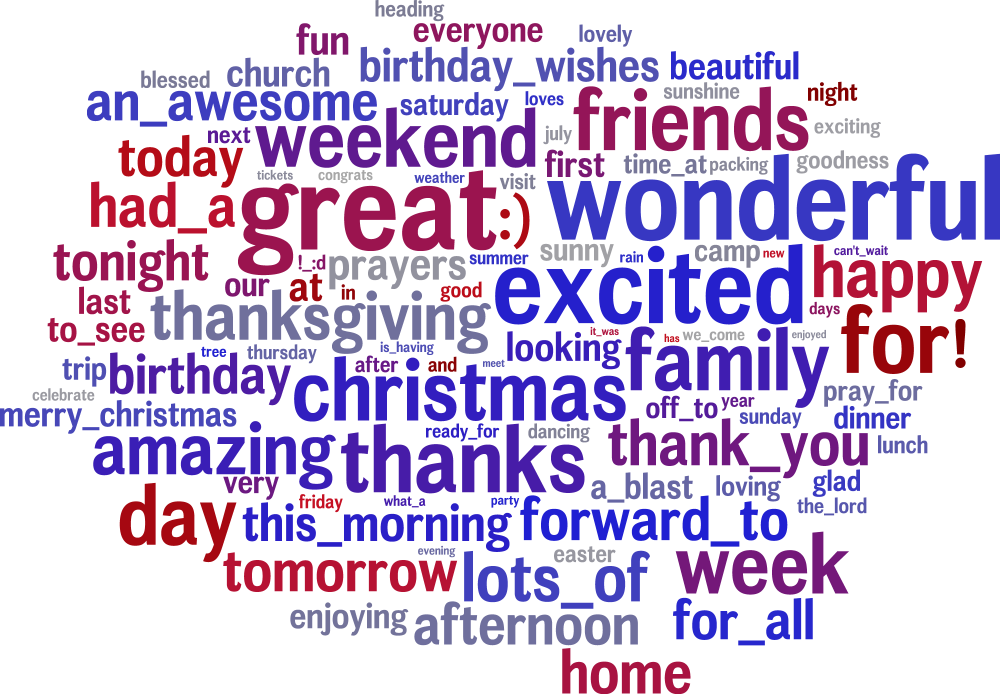


| 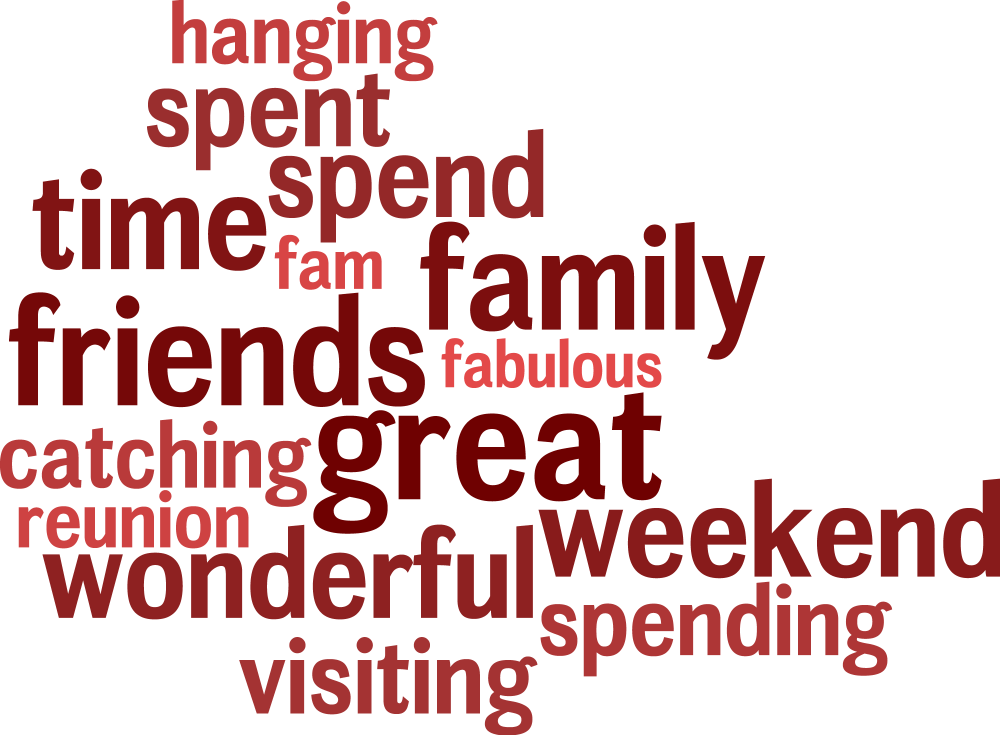 | 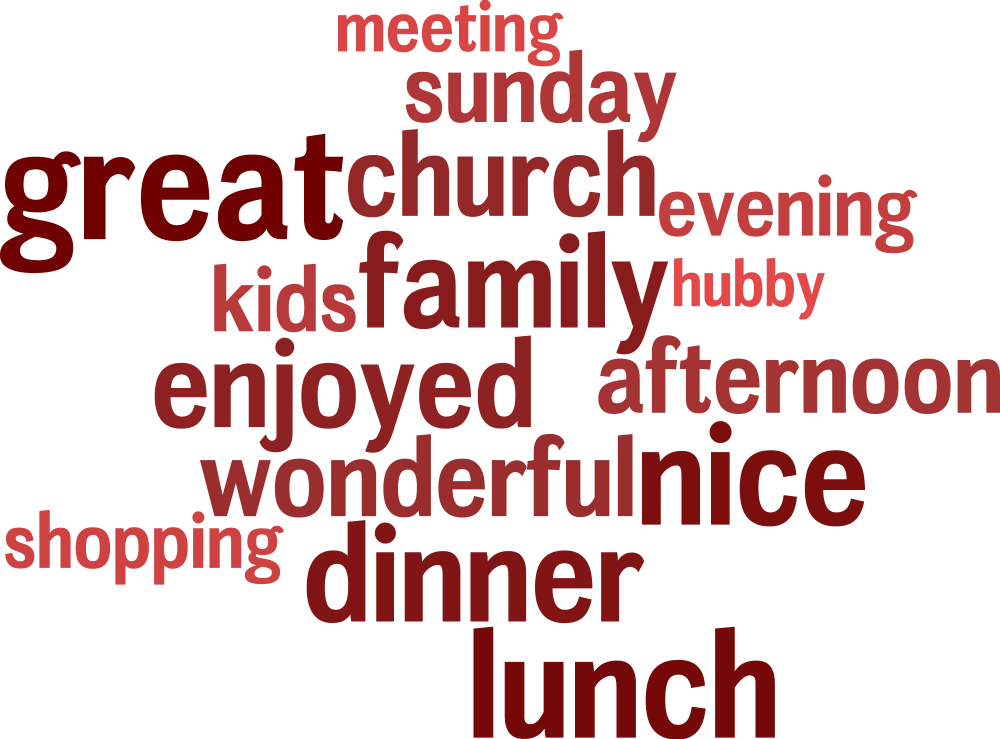 | 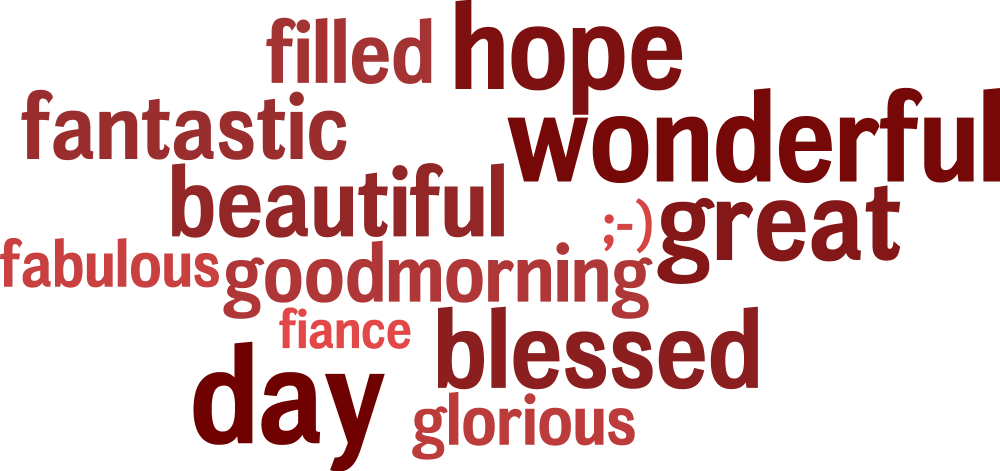 |
| --- | --- | --- |
| 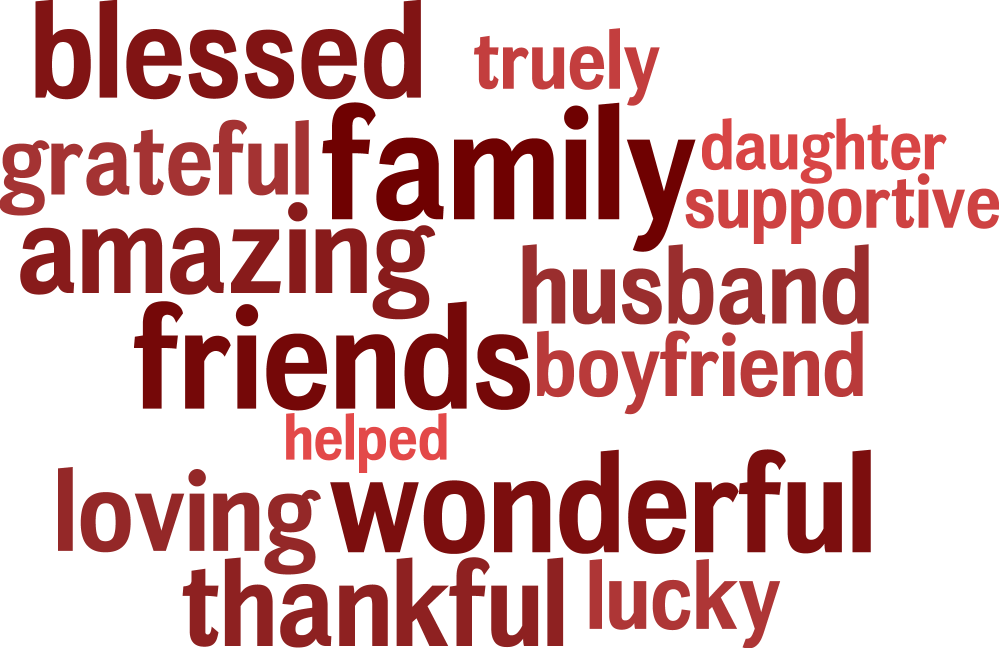 | 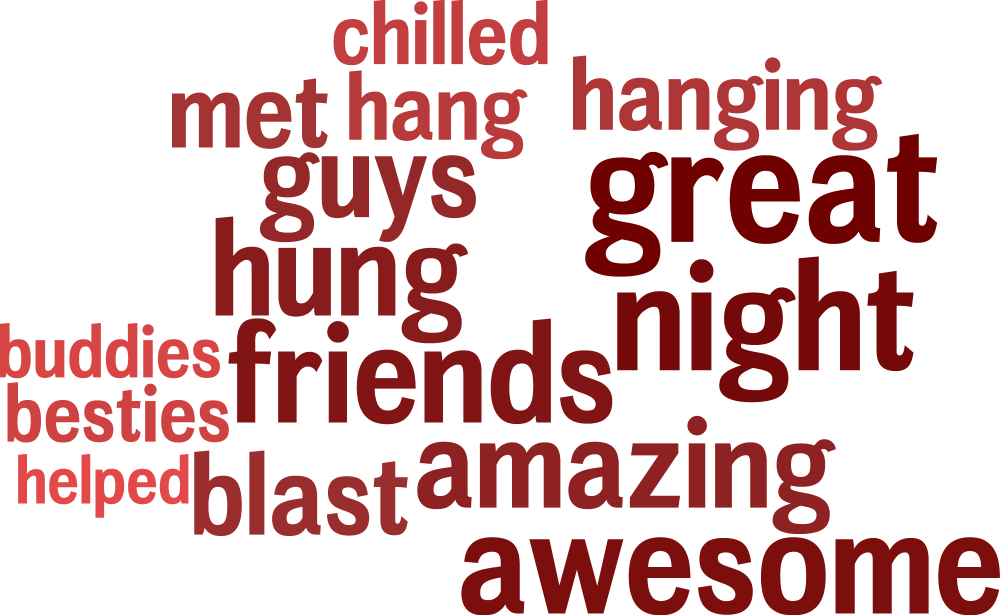 | 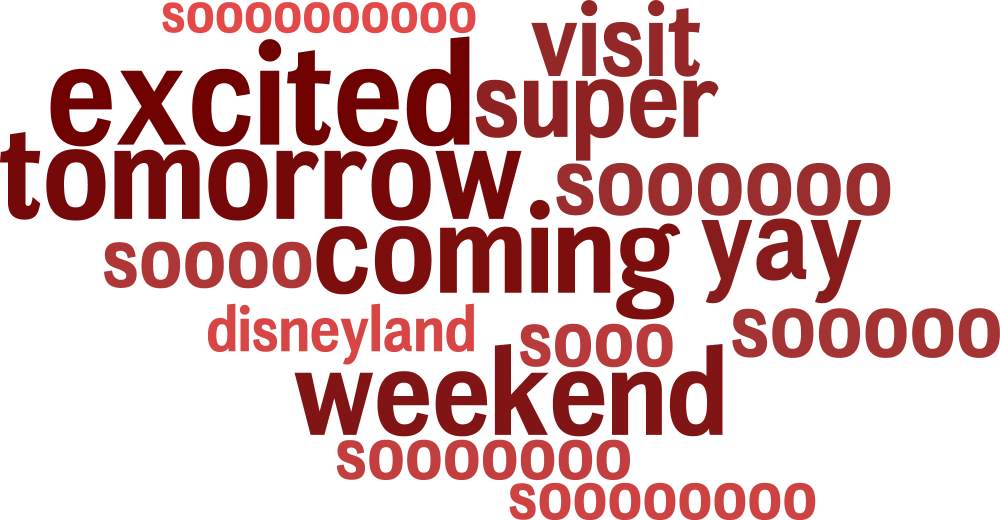 |
| 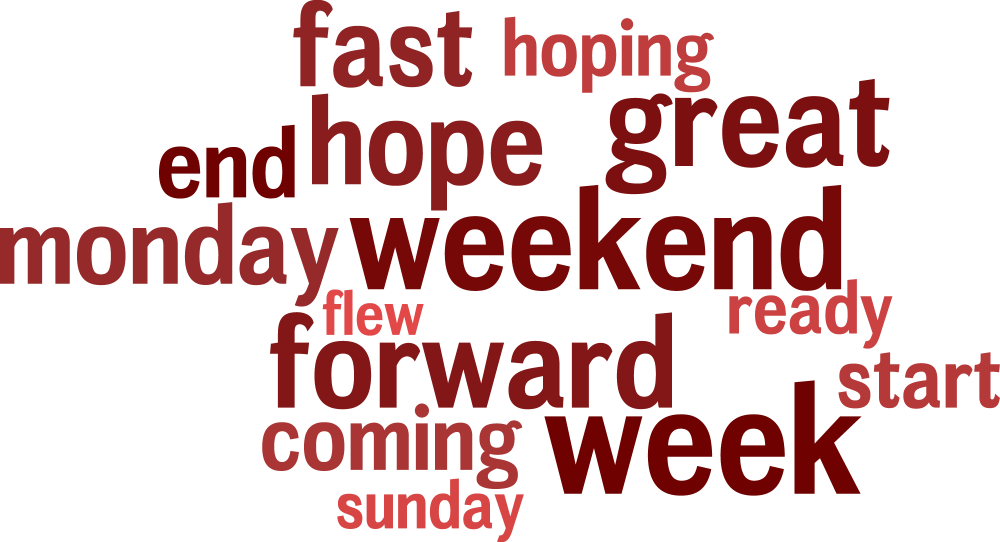 | 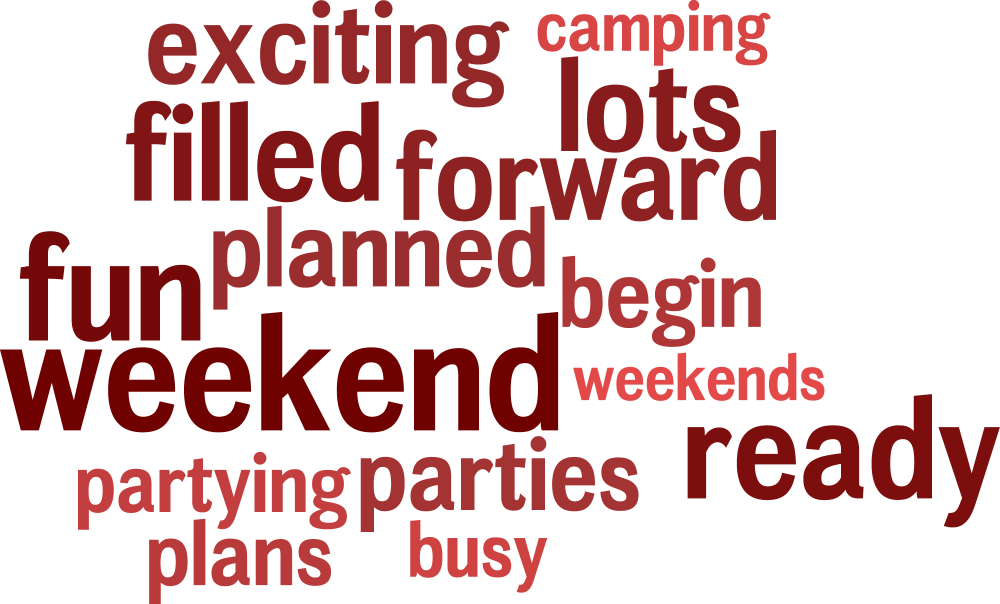 | 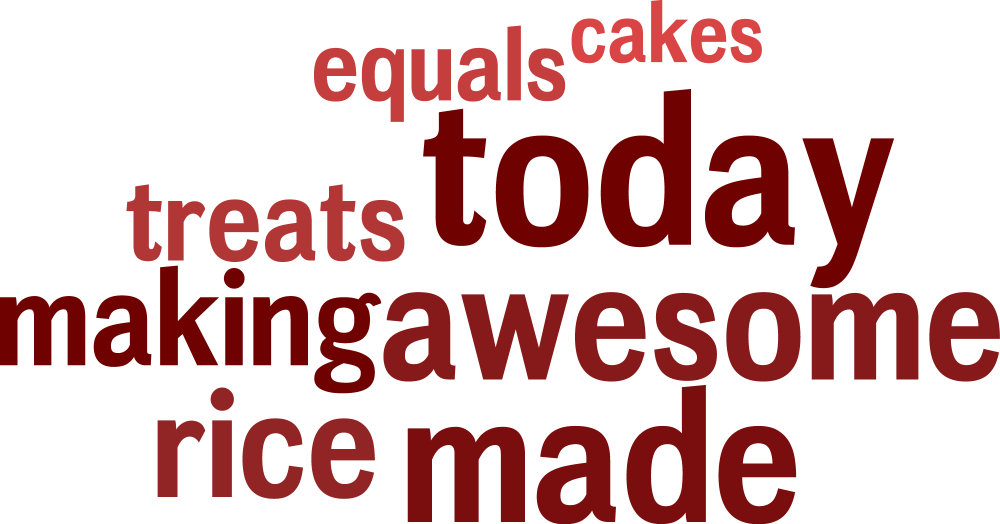 |

*Figure S1.* Positive trust, no controls


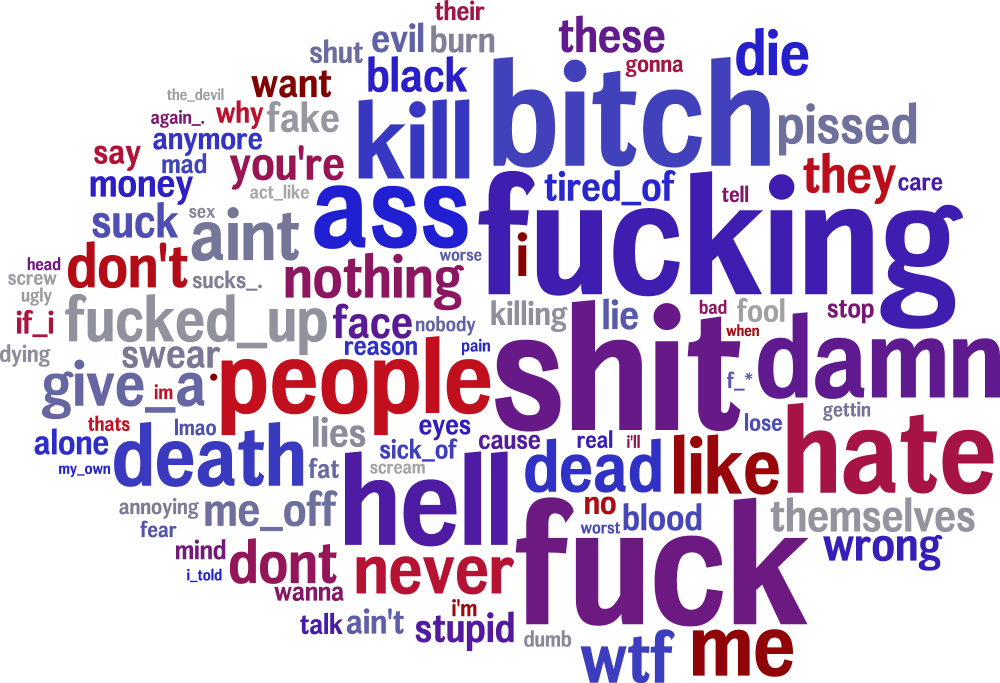


| 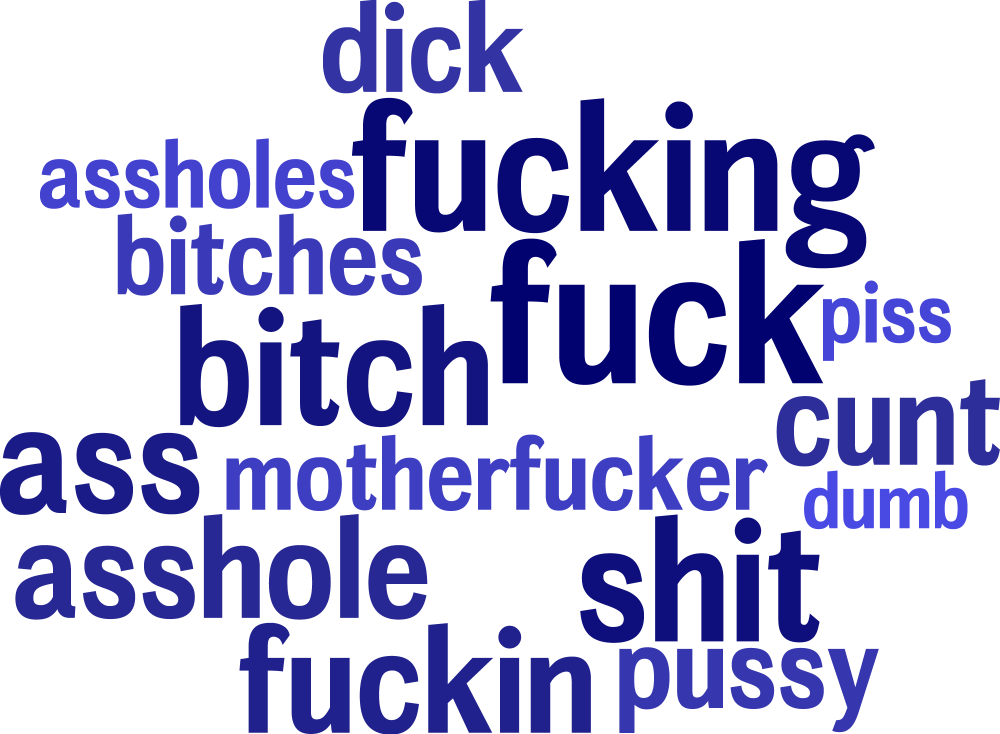 | 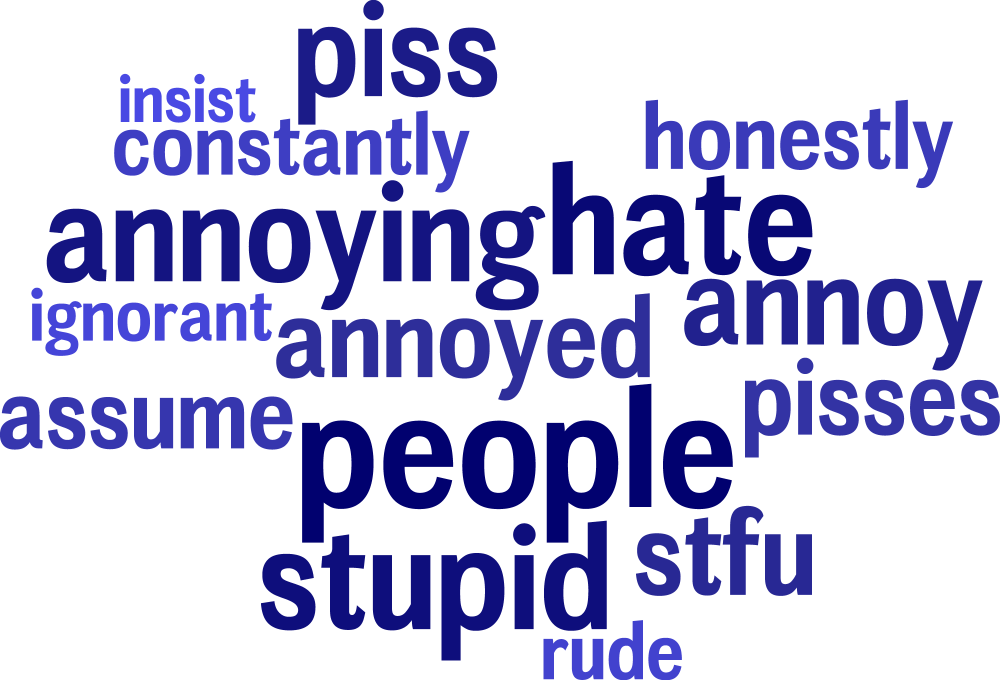 | 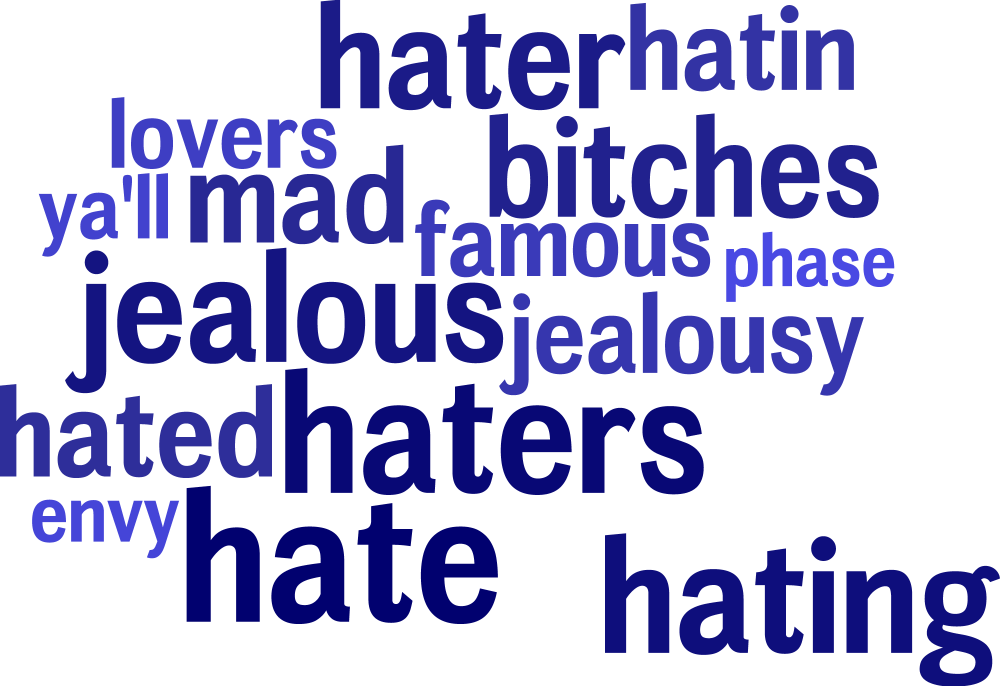 |
| --- | --- | --- |
| 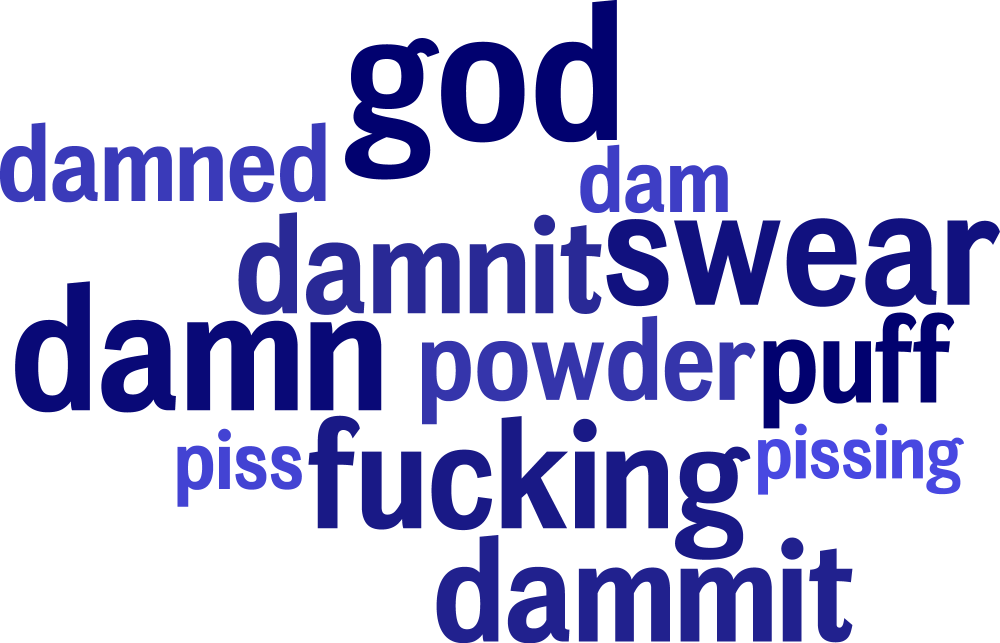 | 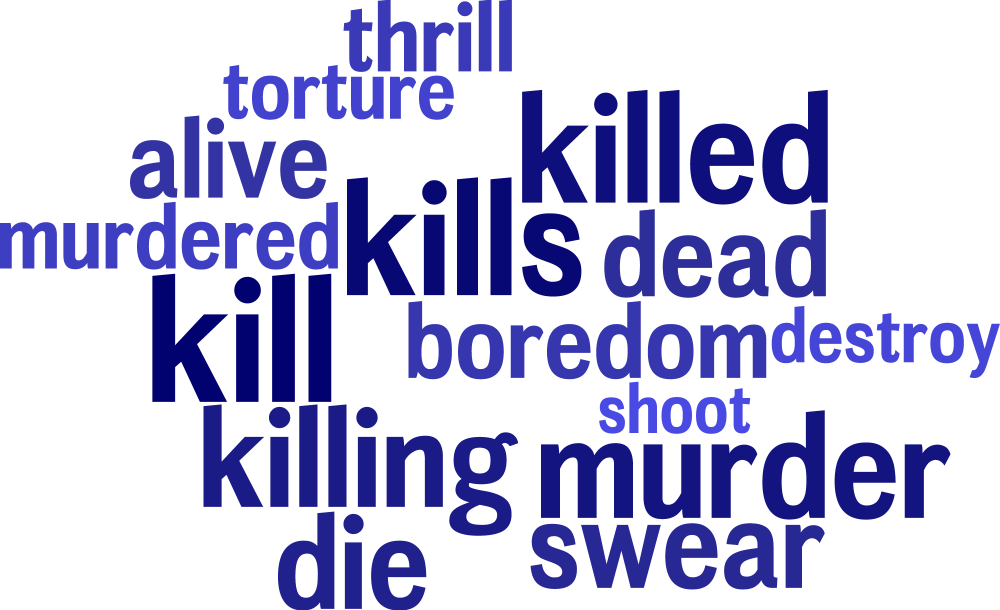 | 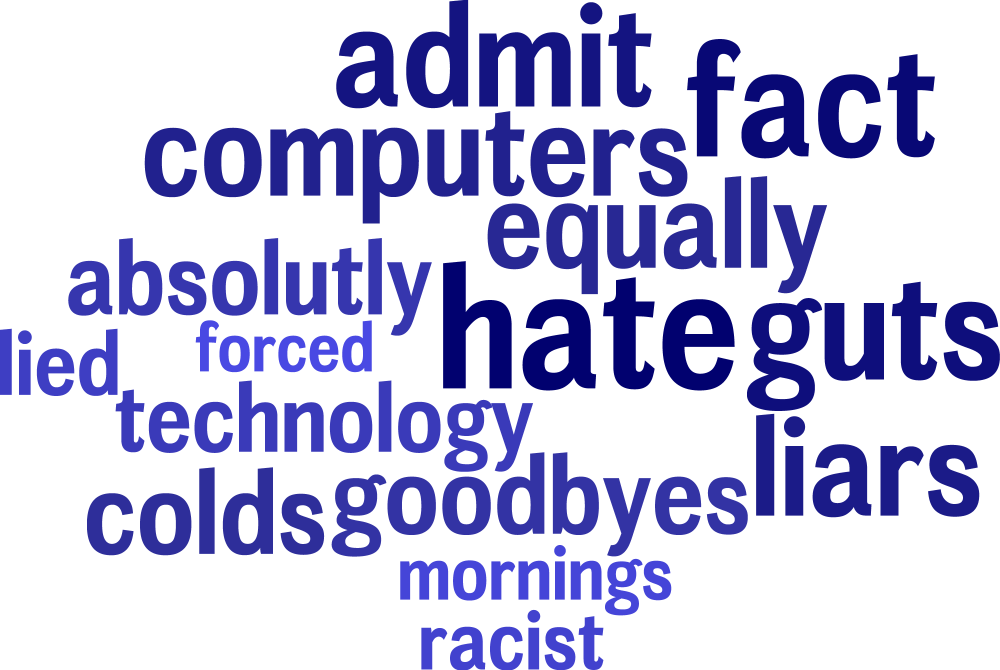 |
| 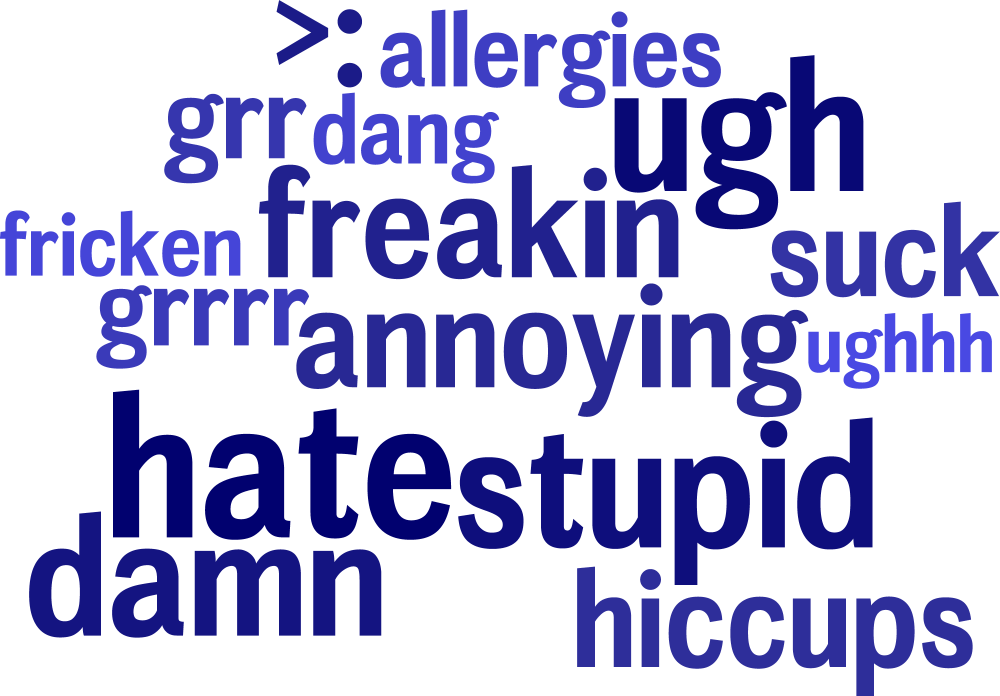 | 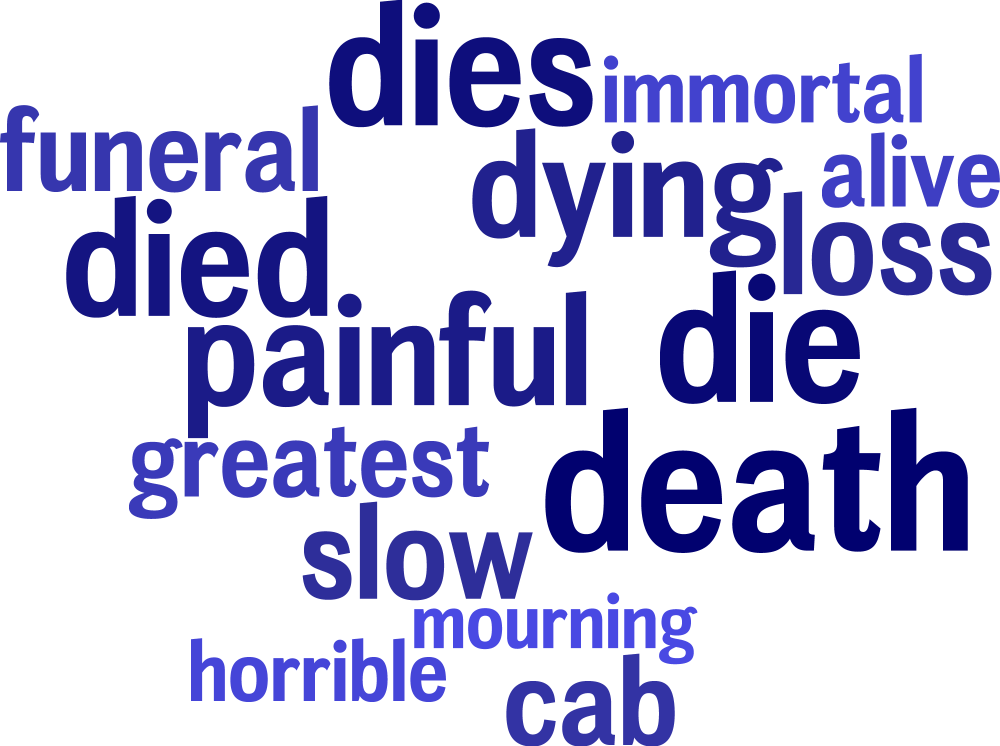 | 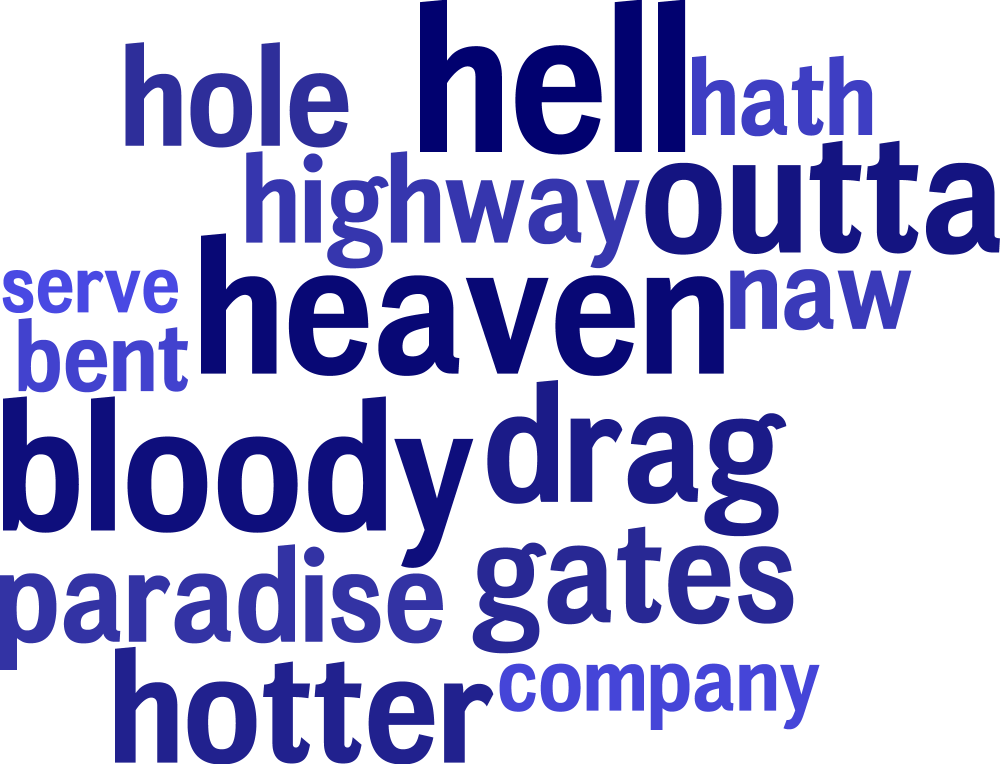 |

*Figure S2.* Negative trust, no controls


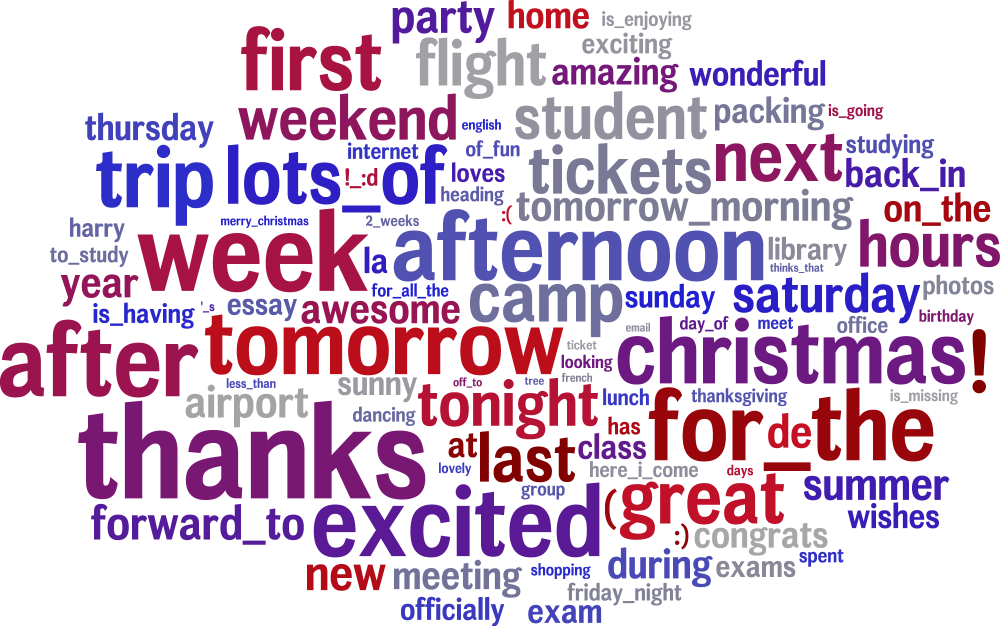


| 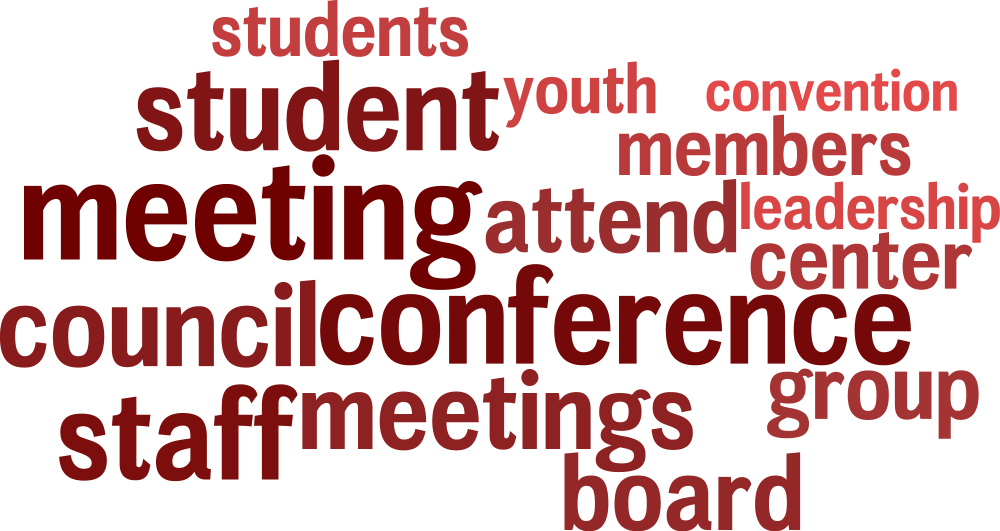 | 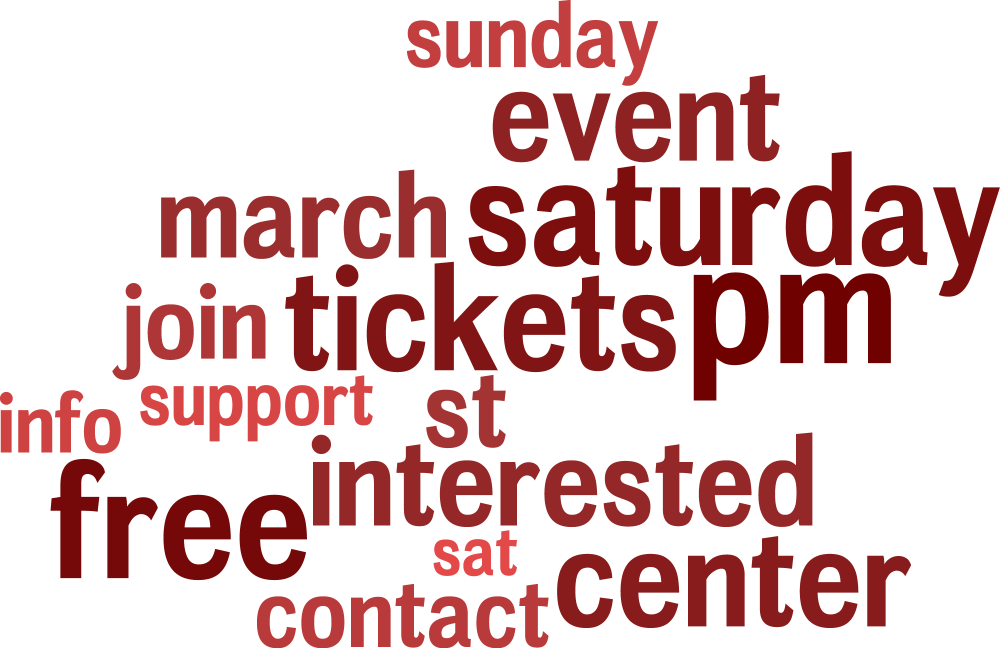 | 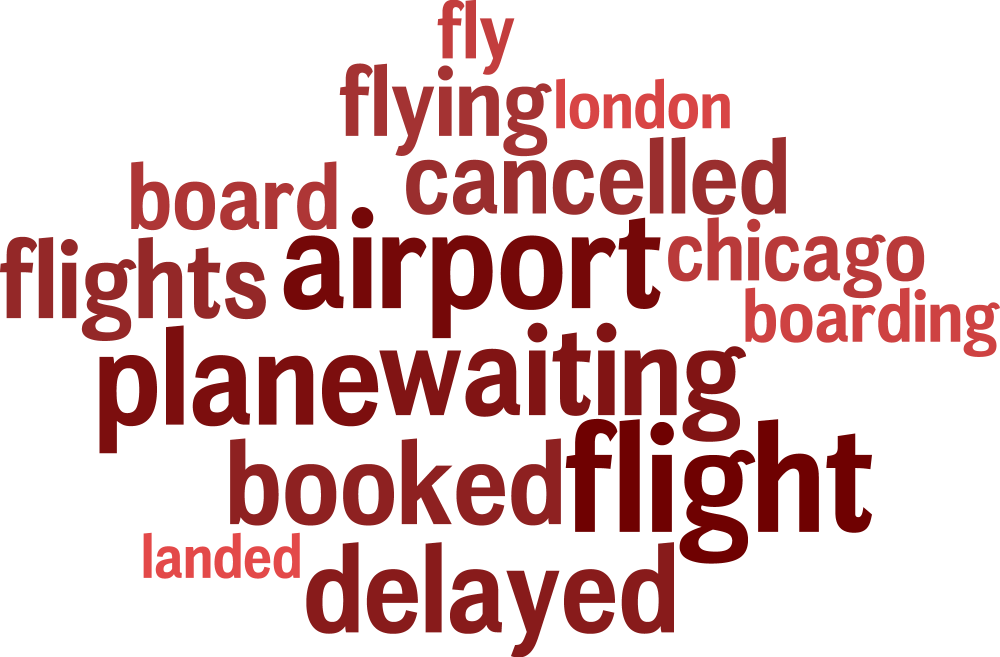 |
| --- | --- | --- |
| 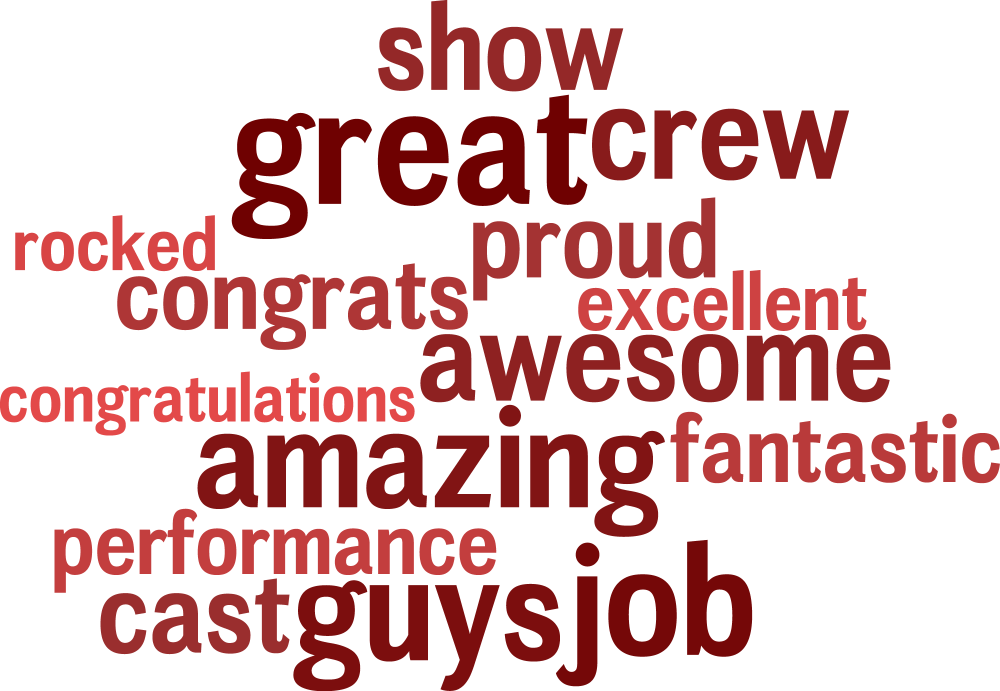 | 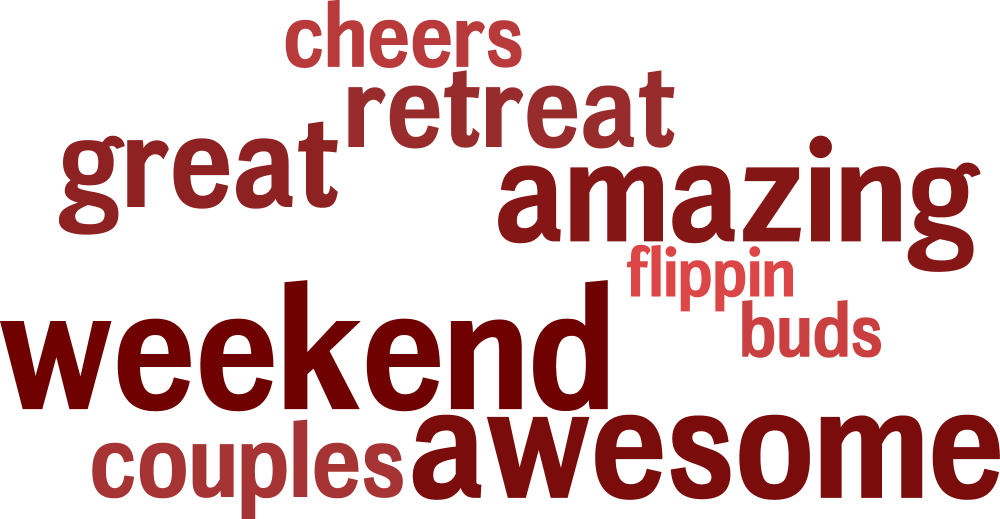 | 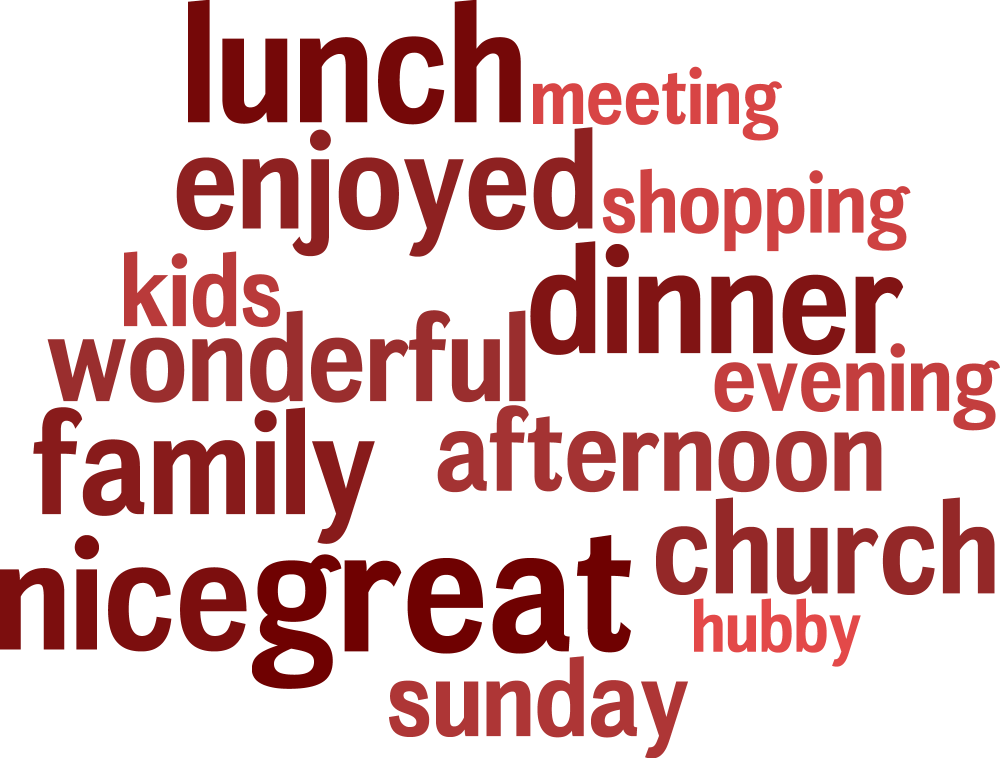 |
| 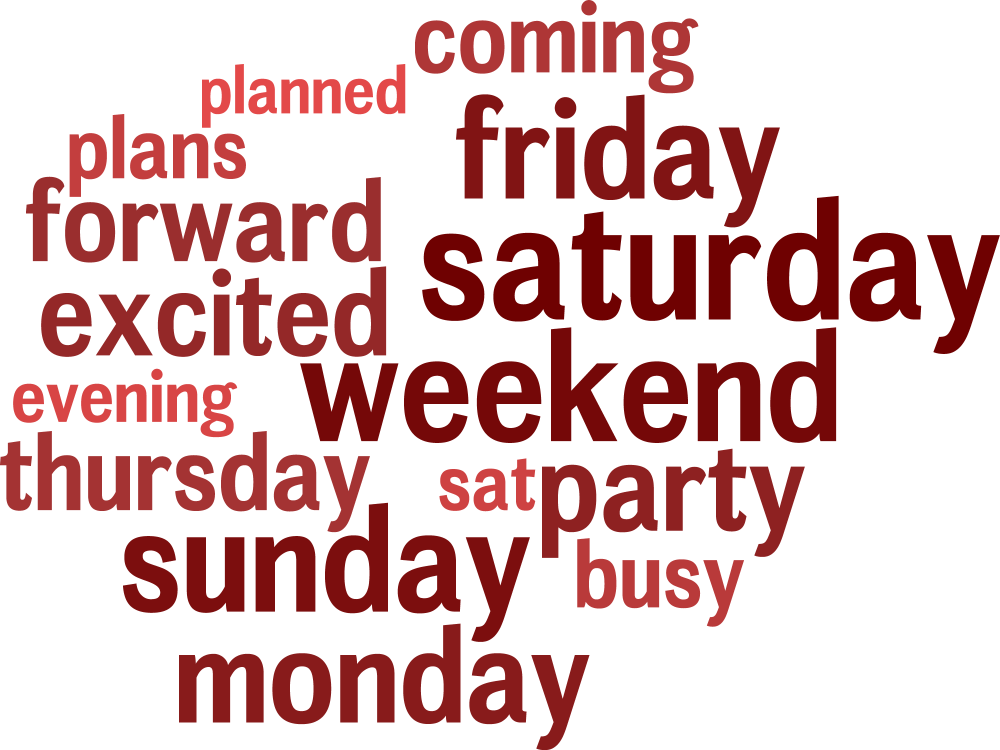 | 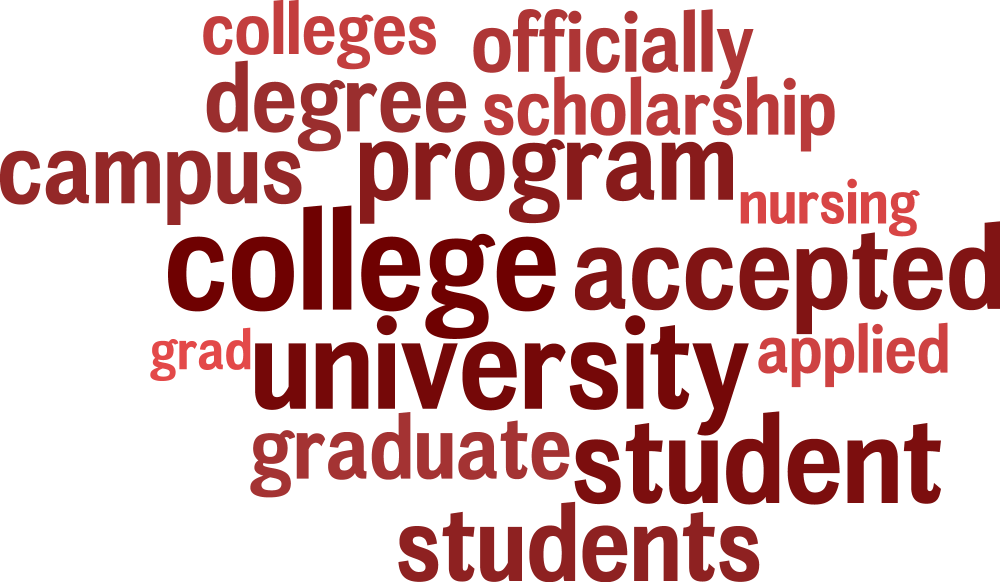 | 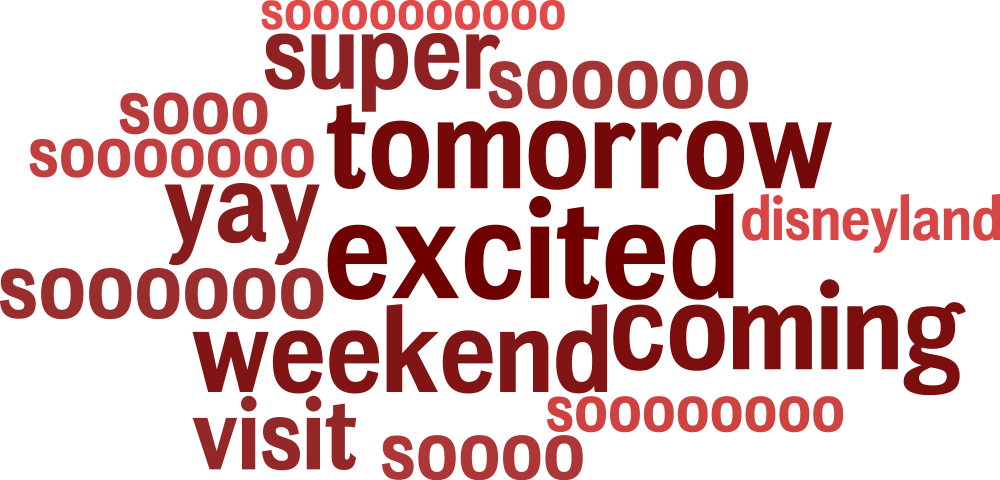 |

*Figure S3.* Positive trust, aggregate agreeableness control (no demographic control)


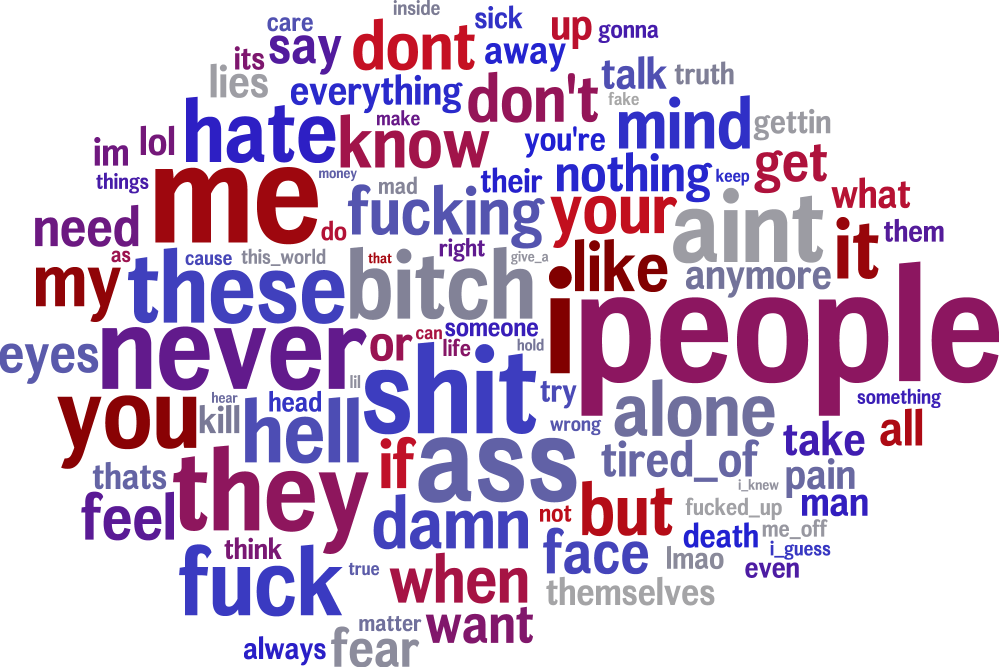


| 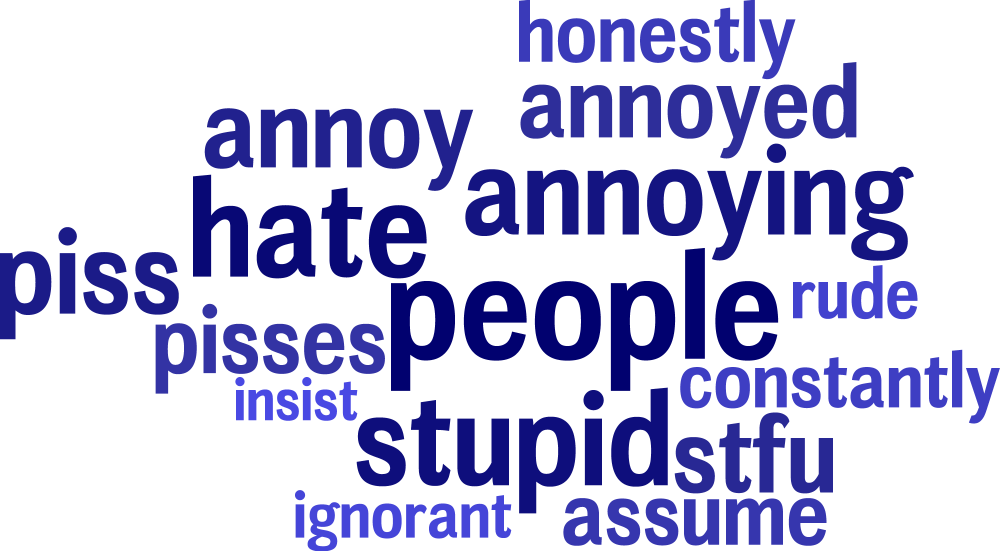 | 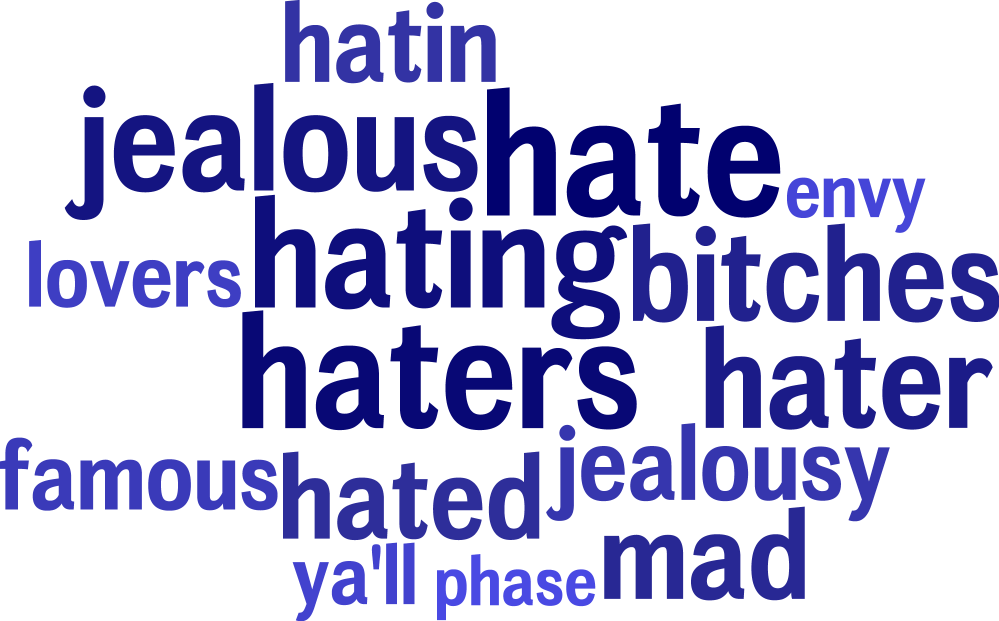 | 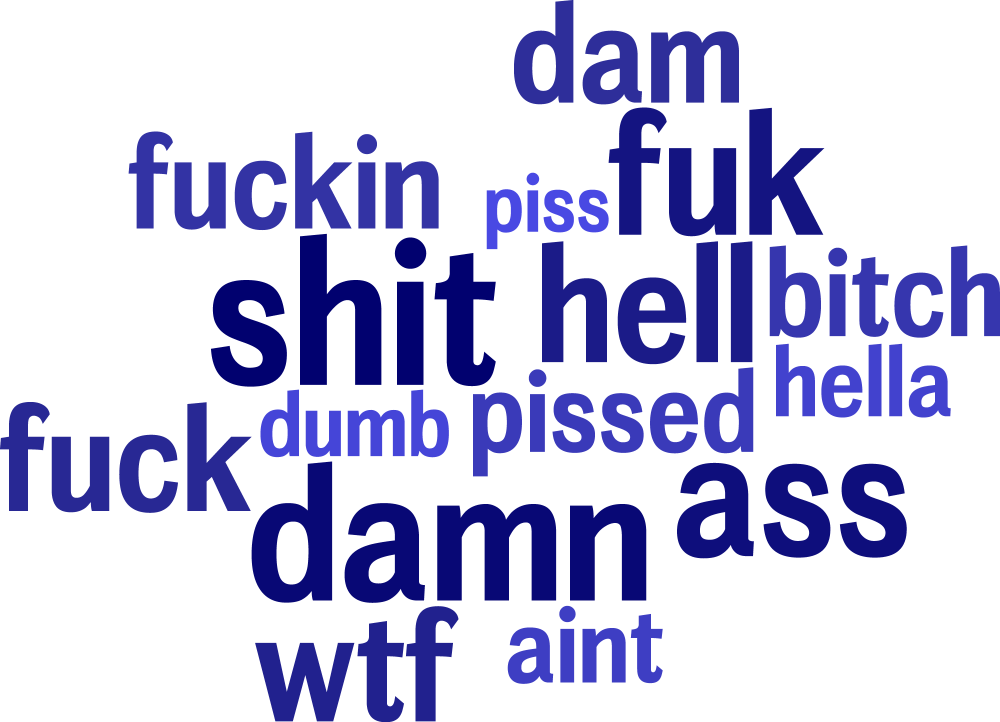 |
| --- | --- | --- |
| 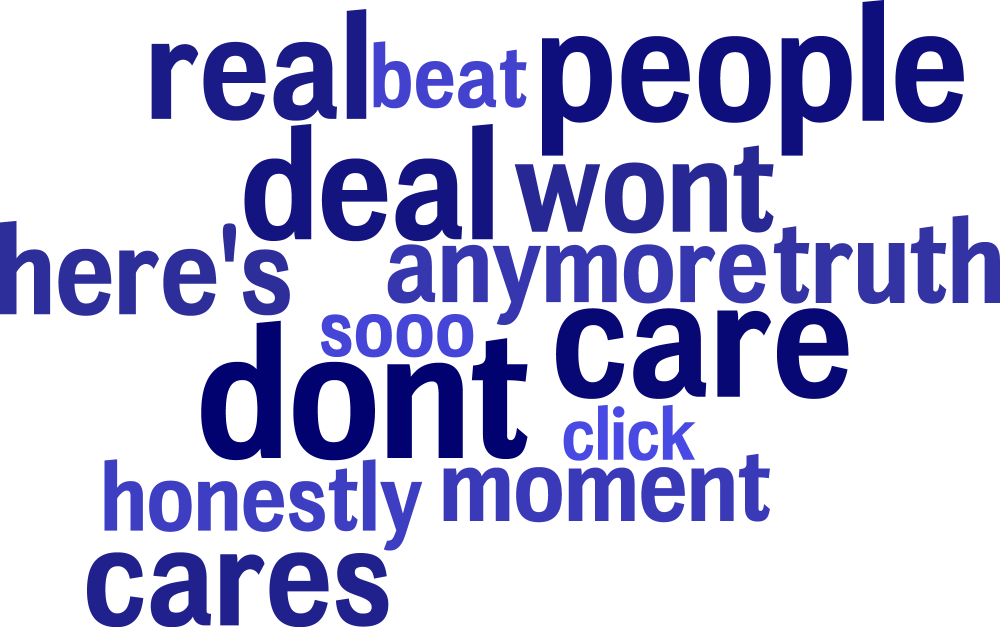 | 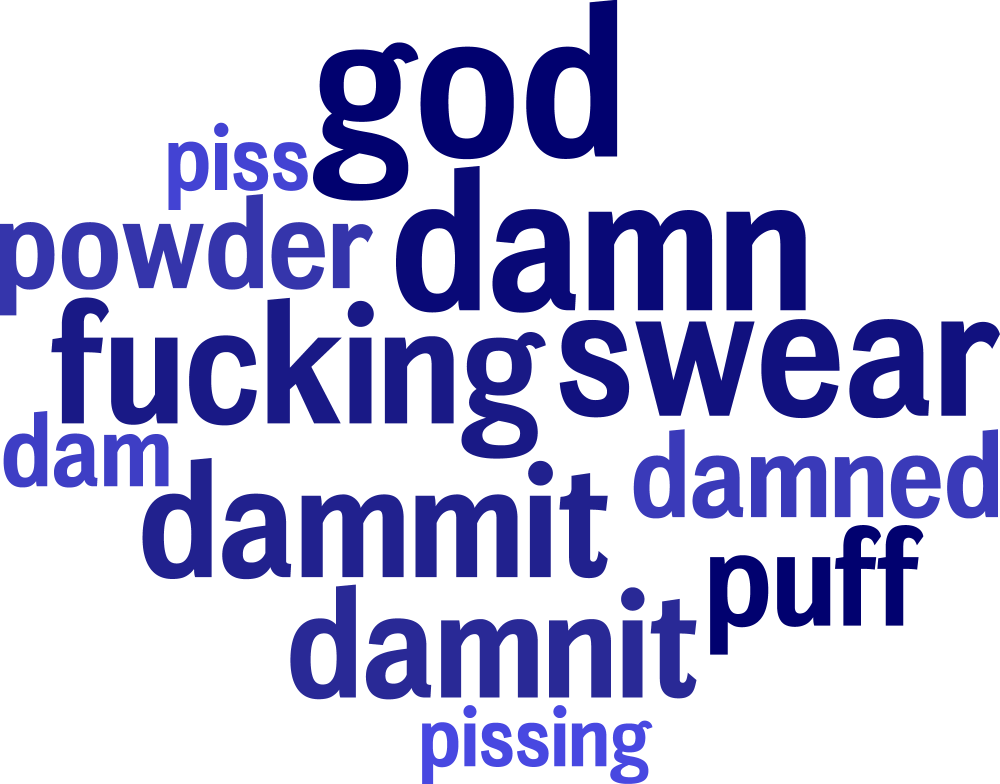 | 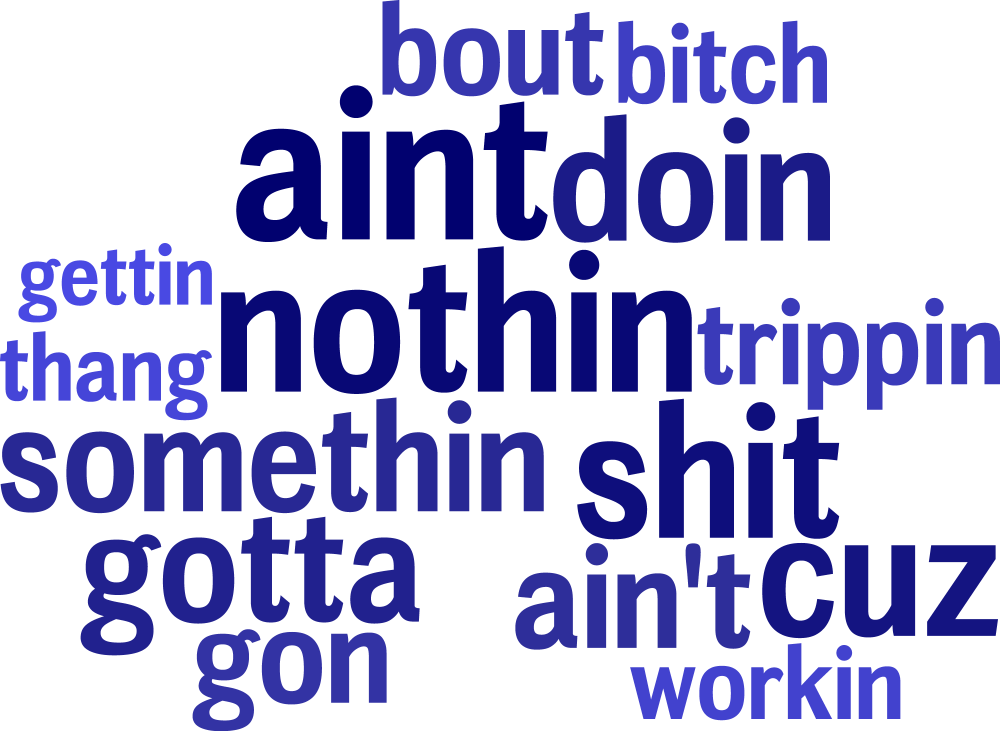 |
| 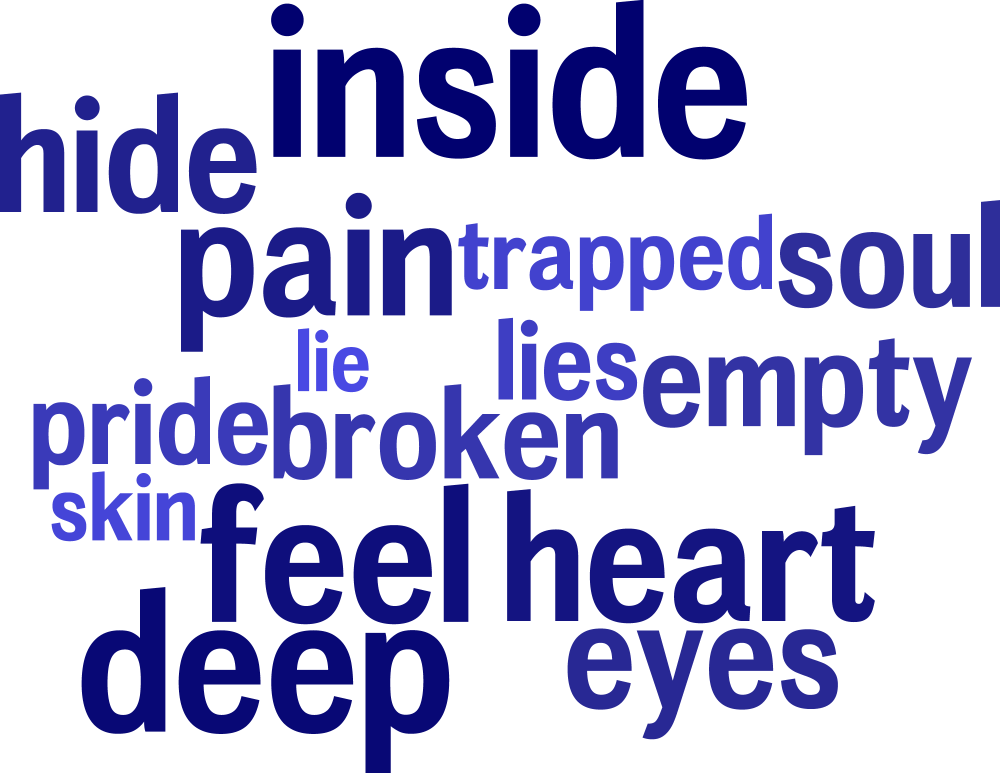 | 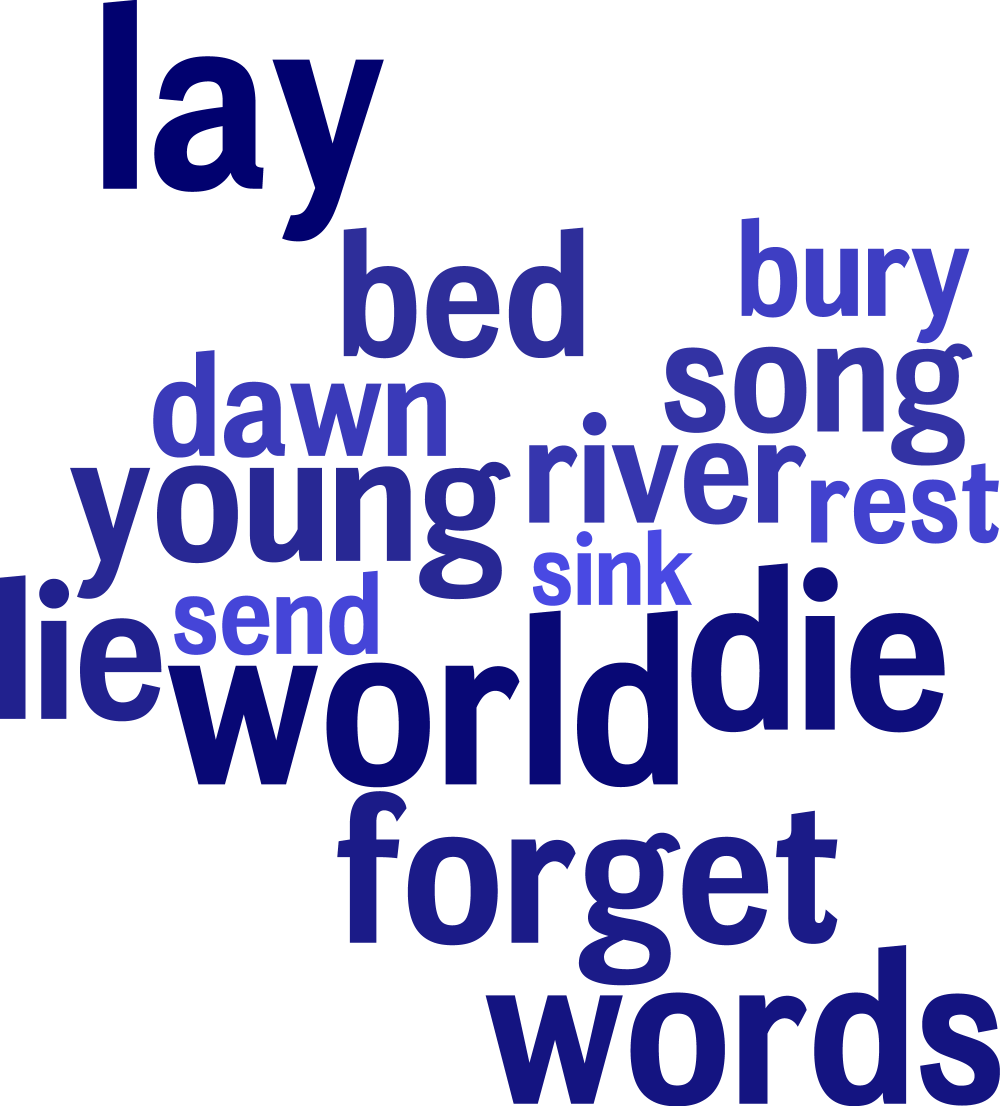 | 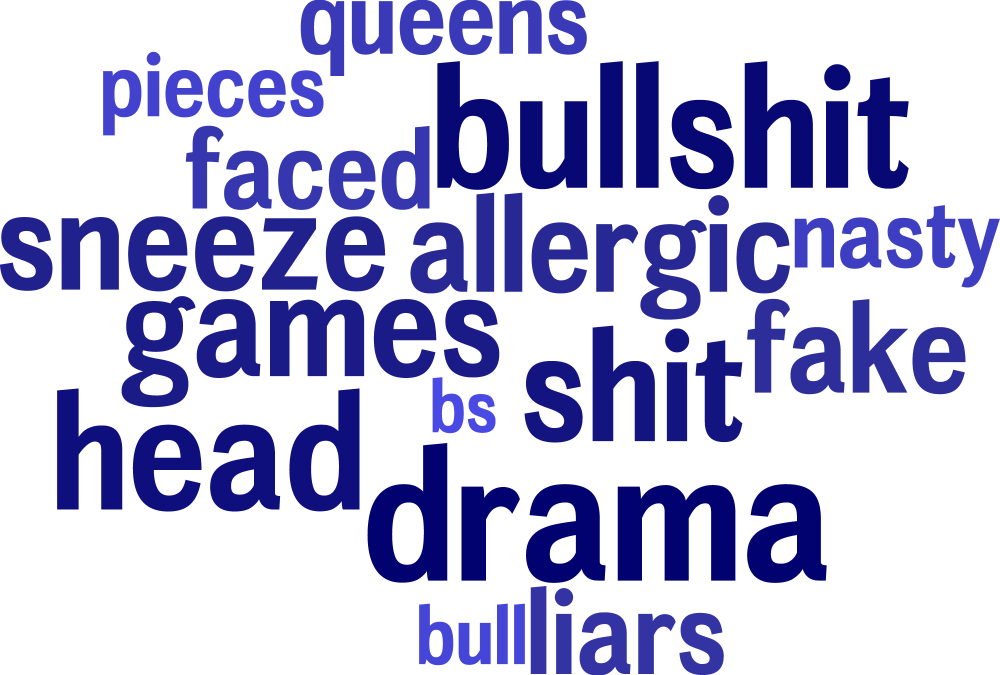 |

*Figure S4.* Negative trust, aggregate agreeableness control (no demographic control)

| **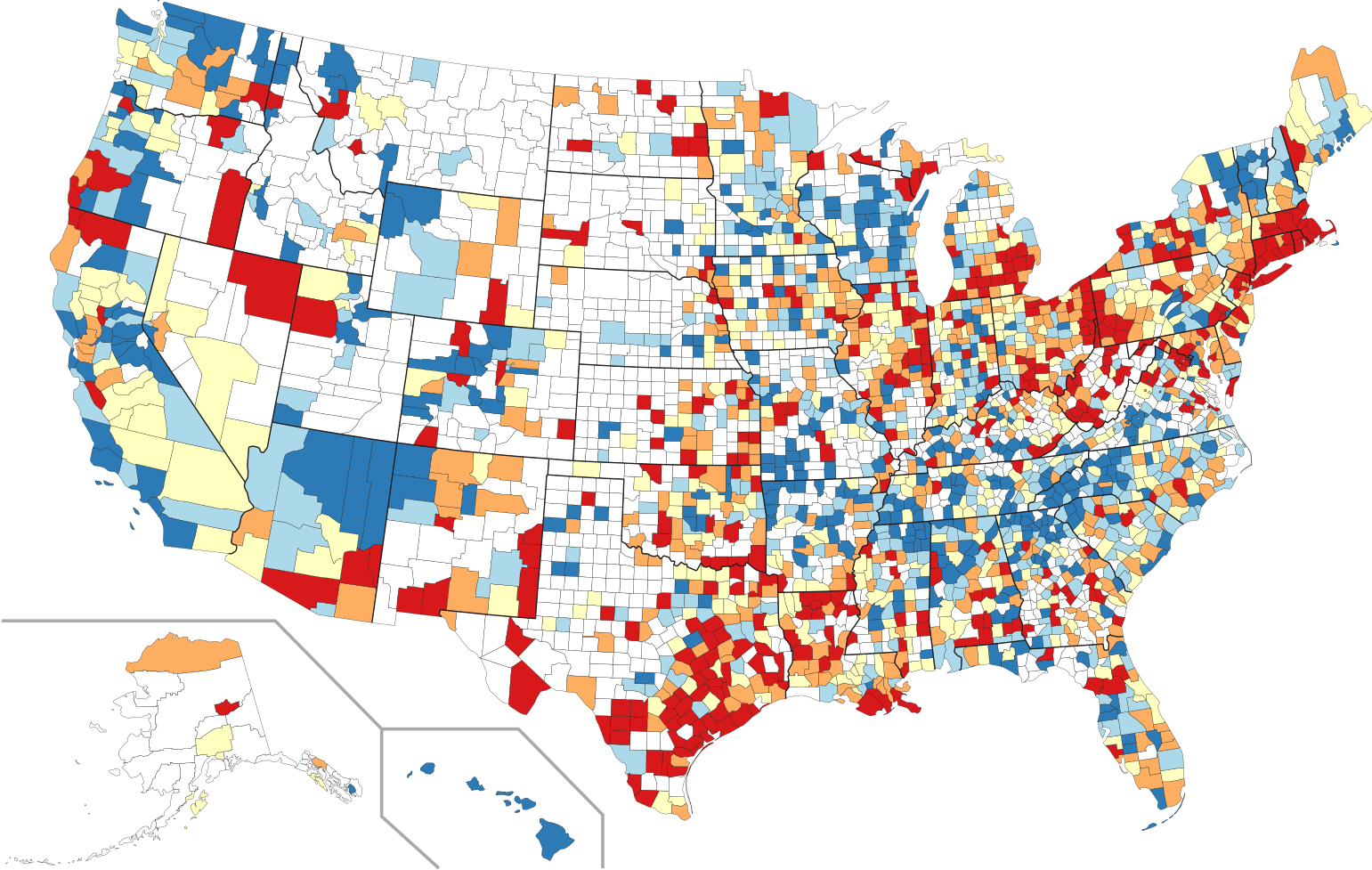** |
| --- |
| ***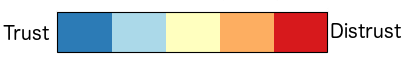*** |

*Figure S5. Levels of trust by U.S. counties controlling for sex, age, race, population, log population density and log income. Blue indicates higher levels of distrust, red indicates lower levels of trust. White counties did not have information available.*

**Table S2**

*County-level correlations between language-based trust and socio-demographics*

|  |  |  |  | Adjusted for log population density | Adjusted for percent bachelor's degree | Adjusted for log median household income |
| --- | --- | --- | --- | --- | --- | --- |
|  |  | Year | *N* | *𝛽* | *𝛽* | *𝛽* |
| *Demographics* | Percent female | 2010 | 2041 | -.11 [-.15, -.06] | -.19 [-.23, -.15] | -.07 [-.11, -.03] |
|  | Median age | 2010 | 2041 | .08 [.03, .12] | .25 [.21, .29] | .07 [.03, .11] |
|  | Log population density | 2010 | 2041 | - | -.26 [-.30, -.22] | -.11 [-.16, -.07] |
| *Socioeconomics* | Log income | 2010 | 2041 | .38 [.34, .41] | .08 [.03, .12] | - |
|  | Percent high school graduates | 2005-09 | 2041 | .53 [.50, .56] | .27 [.23, .31] | .32 [.28, .36] |
|  | Gini | 2010-14 | 2036 | -.16 [-.20, -.11] | -.29 [-.33, -.25] | .04 [-.00, .08]^✝^ |
| *Health and Wellbeing* | Year potential life lost rate | 2012 | 2037 | -.54 [-.57, -.51] | -.34 [-.38, -.31] | -.33 [-.36, -.29] |
|  | Self-rated health, percent fair/poor | 2012 | 1924 | -.50 [-.53, -.46] | -.25 [-.29, -.20] | -.28 [-.32, -.24] |
|  | Percent obese | 2012 | 2041 | -.55 [-.58, -.52] | -.31 [-.35, -.27] | -.41 [-.44, -.37] |
|  | Percent smokers | 2012 | 1832 | -.30 [-.34, -.25] | -.02 [-.06, .03]^✝^ | -.15 [-.19, -.10] |
|  | Percent excessive drinking | 2012 | 1869 | .30 [.26, .35] | .15 [.10, .19] | .15 [.10, .19] |
|  | Life satisfaction | 2009-10 | 1749 | .30 [.26, .34] | .13 [.07, .18] | .20 [.15, .24] |
| *Lifestyle* | Percent married | 2005-09 | 2041 | .26 [.22, .30] | .42 [.38, .46] | .11 [.07, .15] |
|  | Percent separated | 2005-09 | 2041 | -.53 [-.56, -.50] | -.51 [-.54, -.47] | -.41 [-.45, -.37] |
|  | Percent of same-sex households | 2005-09 | 2041 | .23 [.18, .27] | .07 [.03, .11] | .16 [.12, -.20] |
| *Mental health* | Mentally unhealthy days | 2012 | 2016 | -.23 [-.27, -.19] | -.05 [-.09, -.00]^✝^ | -.09 [-.13, -.04] |

*Notes.* Reported standardized beta with 95% confidence intervals in square brackets. All results significant at p < .005 after adjusting for multiple comparisons except ^✝^ not significant. Log population density (except for Turnout), percent of the population with a Bachelor’s degree and log median household income are added as covariates in regression.

**Table S3**

*County-level correlations between language-based trust and religious affiliation*

|  |  |  |  | No adjustments | Adjusted for region | Adjusted for log population density | Adjusted for percent bachelor's degree | Adjusted for log median household income |
| --- | --- | --- | --- | --- | --- | --- | --- | --- |
|  |  | Year | *N* | *r* | *𝛽* | *𝛽* | *𝛽* | *𝛽* |
| *Religion* | Evangelical Protestant | 2010 | 2041 | -.23  [-.27, -.19] | .01  [-.04, .05]^✝^ | -.22  [-.26, -.18] | -.05  [-.09, -.00]^✝^ | -.06  [-.10, -.02] |
|  | Main Protestant | 2010 | 2040 | .13  [.09, .18] | .14  [.09, .18] | .14  [.10, .19] | .16  [.12, .21] | .14  [.09, .18] |
|  | Catholic | 2010 | 1993 | .10  [.05, .14] | -.04  [-.09, .00]^✝^ | .09  [.04, .13] | -.06  [-.10, -.01]^▲^ | -.05  [-.09, -.00]^✝^ |

*Notes.* Reported Pearson *r* and standardized beta with 95% confidence intervals in square brackets. All results significant at p < .001 after adjusting for multiple comparisons except ^▲^ p < .05 and ^✝^ not significant. Region (binary indicator for four Census regions: Northeast, South, West and Midwest), log population density, percent of the population with a Bachelor’s degree and log median household income are added as covariates in regression.

**Table S4**

*County-level correlations between language-based trust and politics*

|  |  |  | Adjusted for log population density | Adjusted for percent bachelor's degree | Adjusted for log median household income |
| --- | --- | --- | --- | --- | --- |
|  | Years | *N* | *𝛽* | *𝛽* | *𝛽* |
| *% Republican votes In Presidential Election* | 2012 | 2032 | .04 [.00, .09]^▲^ | .30 [.26, .34] | .07 [.03, .11] |
|  | 2016 | 2031 | -.04 [-.08, .01]^✝^ | .32 [.28, .36] | .02 [-.02, .07]^✝^ |
| *Trump Vote Gain versus Romney Vote* | 2012-2016 | 2031 | -.24 [-.28, -.20] | .13 [.08, .17] | -.12 [-.16, -.08] |
| *Trump Vote Gain versus Past 4 Republicans* | 2000-2016 | 2028 | -.11 [-.15, -.07] | .28 [.24, .32] | -.01 [-.04, .05]^✝^ |
| *Donation Partisanship* | 2012 | 1665 | .20 [.16, .25] | .02 [-.03, .06]^✝^ | .16 [.12, .21] |
| *Turnout* | 2012 | 2032 | - | .16 [.12, .20] | .21 [.17, .25] |
|  | 2016 | 2031 | - | .27 [.23, .31] | .29 [.25, .33] |

*Notes.* Reported standardized beta with 95% confidence intervals in square brackets. All results significant at p < .01 after adjusting for multiple comparisons except ^▲^ p < .05 and ^✝^ not significant. Log population density (except for Turnout), percent of the population with a Bachelor’s degree and log median household income are added as covariates in regression.
